# Dynamic epigenetic regulation of BCLAF1 splicing in acute myeloid leukemia

**DOI:** 10.1038/s41419-026-08594-4

**Published:** 2026-03-24

**Authors:** Giulia Sgueglia, Crescenzo Massaro, Annamaria Muro, Ida Lettiero, Erika D’Agostino, Gregorio Favale, Nicla Simonelli, Nunzio Del Gaudio, Vincenzo Carafa, Tommaso De Marchi, Dante Rotili, Sergio Valente, Antonello Mai, Gianluca Sbardella, Mariacarla De Simone, Lucia Altucci, Carmela Dell’Aversana

**Affiliations:** 1 Department of Precision Medicine, University of Campania “L. Vanvitelli”, Naples, Italy; 2 Department of Pathology, Cancer Center Amsterdam, Amsterdam UMC, Vrije Universiteit Amsterdam, Amsterdam, the Netherlands; 3 Link Campus University, Rome, Italy; 4 BIOGEM, Ariano Irpino, Italy; 5 Department of Clinical Sciences Lund, Division of Oncology, Lund University, Lund, Sweden; 6 Department of Science, “Roma Tre” University, Rome, Italy; 7 Biostructures and Biosystems National Institute (INBB), Rome, Italy; 8 Department of Drug Chemistry and Technologies, Sapienza University of Rome, Rome, Italy; 9 Department of Pharmacy, Epigenetic Med Chem Lab, University of Salerno, Fisciano, Italy; 10 Division of Hematology, AORN Cardarelli, Naples, Italy; 11 Medical Epigenetics program, Vanvitelli Hospital, Naples, Italy; 12 Institute of Endotypes in Oncology, Metabolism, and Immunology ‘G. Salvatore’ (IEOMI) -National Research Council (CNR), Naples, Italy; 13 Department of Medicine and Surgery, LUM University, Casamassima, Italy

**Keywords:** Cancer genetics, Epigenetics, Oncogenesis, Mechanisms of disease

## Abstract

Dysregulation of alternative splicing is increasingly associated with cancer development and tumor progression. BCL2-associated transcription factor 1 (BCLAF1) is involved in a wide range of biological processes and it is continuously being investigated due to its intricate function in tumorigenesis and drug resistance. In acute myeloid leukemia (AML) cell lines, we identified two distinct, unbalanced isoforms of BCLAF1: the full-length isoform, which exhibits oncogenic properties, and the short-length isoform, which seems to act as a tumor suppressor. Treatment with specific epidrugs can re-establish the physiological balance of full- and short-length isoforms, restoring their correct equilibrium. Our results suggest the existence of a newly identified mechanism underlying the regulation of BCLAF1 splicing orchestrated, at least in part, by the interplay between HDAC1 and DNMT3A, and directly correlated with the healthy or cancerous state of hematopoietic cells. Our findings shed light on a novel regulatory axis in AML and highlight the potential of epidrugs to restore normal splicing patterns, paving the way for innovative therapies.

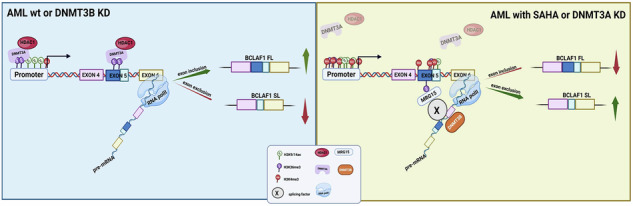

## Introduction

Altered regulation of alternative splicing is a critical event in the generation and development of many types of cancer [1]. Frequently, the alternative splicing mechanism leads to an altered epigenetic chromatin asset, and epigenetic changes can affect the modulation of splicing; conversely, splicing can modulate the expression of various epigenetic factors, highlighting the fact that these two processes are strictly interconnected [2]. One of the major causes of altered splicing is the result of changes in histone epigenetic marks [2]. Such modifications may modify RNA polymerase II processivity and splicing factor recruitment. One of the main histone modifications associated with alternative splicing is H3K36me3, enriched only in the exon of actively transcribed genes and probably involved in RNA polymerase II elongation [3, 4]. Also, H3K4me3 modification is linked to alternative splicing, affecting enzyme binding activity of DNA methyltransferases (DNMTs) [2]. DNMT3A is commonly mutated in acute myeloid leukemia (AML) patients (20% of adult cases) and can interact with splicing components, impacting the modulation of global alternative splicin Dysregulation of histone deacetylases (HDACs) plays a crucial role in hematological cancers, as they are often overexpressed in leukemias, particularly in AML with fusion proteins such as AML1-ETO and PML-RARα [5]. The influence of HDACs in the regulation of splicing both physiologically and pathologically is widely reported in literature [3]. Specifically, HDAC1 is involved in the control of alternative splicing [6], although the exact functional role of this enzyme is not yet characterized.

Bcl-2-associated transcription factor 1 (BCLAF1) is a multifunctional transcriptional factor involved in several biological processes, both in physiological conditions, such as muscle cell proliferation and differentiation [7], and in pathological contexts, including tumor progression and drug/chemotherapy resistance [8, 9]. In physiological conditions, BCLAF1 exerts pro-apoptotic activity by forming a complex with PKCδ in response to DNA damage, thereby promoting TP53-mediated apoptosis [9]. Through the formation of a complex with BRCA1, BCLAF1 regulates messenger RNA (mRNA) splicing, likely generating mRNAs essential for DNA damage repair, thus functioning as a tumor suppressor complex [10]. BCLAF1 also interacts with BACH1and BRCA1 forming a complex and initiating DNA damage response [10–12]. Conversely, the role of BCLAF1 in driving cancer progression is well reported, especially in AML as well as in colorectal cancer, bladder cancer, lung cancer, hepatocellular carcinoma, and gastric cancer [13–15]. BCLAF1 is overexpressed in all these cancer types, and is highly expressed in AML, in contrast to its targeting microRNA (miRNA), miR-194-5p. This miRNA targets BCLAF1 and inhibits its translation in AML. Furthermore, the resulting decrease in BCLAF1 expression causes G1 cell cycle arrest and activation of caspase 9-dependent apoptosis [16].

The *BCLAF1* gene is located on chromosome 6 and potentially encodes up to 17 predicted different isoforms (Ensembl database, 2023). However, the functional significance of BCLAF1 isoforms has only been validated in colorectal cancer. Three different transcripts were validated: the full-length (FL) isoform with all 13 exons, a short-length (SL) isoform lacking the majority (519 bp) of exon 5, and an isoform lacking only the entire exon 11 [17]. The FL isoform (with exon 5a) promotes tumor progression, proliferation, and migration and is overexpressed in colon cancer cell lines while the SL isoform (without exon 5a), is downregulated and seems to act as a tumor suppressor. The alternative splicing of BCLAF1 isoforms in colorectal cancer is driven by serine and arginine-rich splicing factor 10 (SRSF10), by promoting exon 5a inclusion, generating the oncogenic FL isoform. The intricate balance between these two “antagonistic” isoforms seems to be closely associated with tumorigenesis and the aggressiveness of cancer.

Our study provides a comprehensive molecular and functional characterization of the FL and truncated isoforms of BCLAF1 in AML, as well as an in-depth insight into the epigenetic regulating this aberrant balance. Our findings highlight the potential therapeutic value of drugs or epidrugs capable of modulating alternative splicing to reverse splicing in favor of the SL isoform of BCLAF1. This approach could represent a promising strategy for overcoming chemotherapy resistance associated with BCLAF1 overexpression, improving treatment outcomes for patients with resistant tumors.

## Materials and methods

### Biological resources

U937, K562, and NB4 cell lines were grown in RPMI 1640 medium (EuroClone, ECB2000) supplemented with 10% heat inactivated FBS (EuroClone, ECS5000L), 1% glutamine (MicroGem, TCL012), 1% penicillin/streptomycin (EuroClone, ECB3001D), and 0.01% amphotericin (EuroClone, ECM0009D). U937 shDNMT3A and U937 shDNMT3B cells generated by retroviral transduction were grown in RPMI complete medium with 1 μg/mL puromycin. HEK293FT cell lines were grown in DMEM (EuroClone, ECM0101) supplemented with 10% heat inactivated FBS (EuroClone, ECS5000L), 1% glutamine (MicroGem, TCL012), 1% penicillin/streptomycin (EuroClone, ECB3001D), and 0.01% amphotericin (EuroClone, ECM0009D). CD34^+^ progenitors and peripheral blood AML samples were obtained in accordance with ethical guidelines approved by the Ethics Committee of the University of Campania “Luigi Vanvitelli” Hospital (Prot. number: 296).

### RNA isolation, reverse transcription, and RNA nascent synthesis

Total RNA was isolated using TRIzol™ Reagent (Invitrogen Life Technologies) following the manufacturer’s instructions. Quantification and purification of total RNAs was performed using a NanoDrop 2000 spectrophotometer (ThermoFisher Scientific). RNA (1 μg) was converted to cDNA by reverse transcription using a SuperScript VILO cDNA Synthesis Kit (Invitrogen). A Click-iT™ Nascent RNA Capture Kit (ThermoFisher Scientific) was used for RNA nascent validation according to the manufacturer’s protocol.

### RNA immunoprecipitation (RIP)

RIP was performed using an EZ-Magna RIP® RNA-Binding Protein Immunoprecipitation Kit (Sigma-Aldrich) according to the manufacturer’s instructions. Briefly, U937 wt cells were cultured in RPMI supplemented with 10% FBS at 37 °C and treated for 24 h with 5 µM SAHA; U937-shDNMT3A clone cells were cultured in RPMI supplemented with 10% FBS at 37 °C and treated for 48 h with 2 µg/mL of doxycycline. For immunoprecipitation, 5 µg of anti-SRSF10 (Atlas Antibodies, HPA053831) and anti-DNMT3B (Santa Cruz Biotechnology, sc-376043) were used. The isolated RNAs were used for PCR analysis on BCLAF1 isoforms. The primers used are listed in Table 2.

### Statistical analysis

All experiments were performed in biological triplicate. Statistical analysis was performed using GraphPad Prism 7 software. Statistical p-values were analyzed using a two-way ANOVA, and significance was defined as a p-value of less than 0.05, with the exact degree of significance indicated by asterisks in the figures. (* = *p* < 0.05, ** = *p* < 0.01, *** = *p* < 0.001, **** = *p* < 0.0001).

## Results and Discussion

### Differential expression and epigenetic regulation of BCLAF1 isoforms in AML

**Fig. 1:**
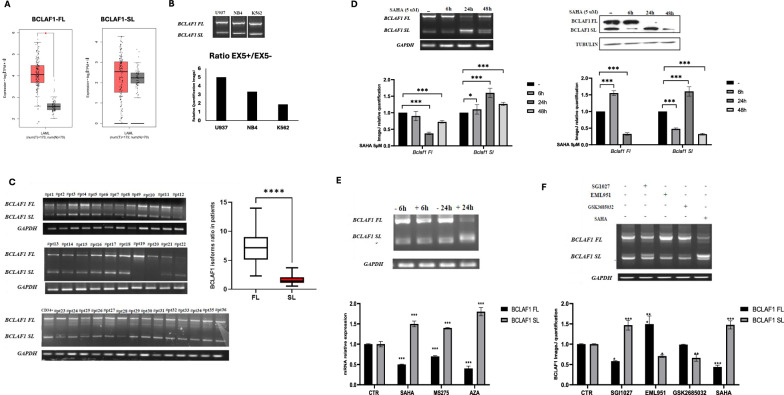
BCLAF1 isoform expression in acute myeloid leukemia (AML). **A** GEPIA analysis on full-length (FL) and short-length (SL) isoforms of BCLAF1 in AML patients compared to healthy controls. **B** PCR analysis of FL and SL isoform expression in three leukemia cell lines (U937, K562, NB4). **C** PCR analysis performed on our cohort of 36 AML patients and one CD34⁺ healthy donor. **D** PCR and Western blot analyses of BCLAF1 expression in U937 cells treated with SAHA (5 µM) for 6, 24, and 48 h. **E** PCR analysis of nascent RNA in U937 cells after 6 and 24 h of SAHA treatment (5 µM). **F** PCR analysis of BCLAF1 expression in U937 cells treated for 24 h with SAHA (5 µM), EML951 (5 µM), SGI-1027 (10 µM), and GSK3685032 (10 µM). Values are presented as the mean ± SEM. Significance was defined as a *p*-value < 0.05, indicated by asterisks in the figures (* = *p* < 0.05, ** = *p* < 0.01, *** = *p* < 0.001, **** = *p* < 0.0001).

We were the first to identify aberrant *BCLAF1* expression in AML, elucidating its critical role as a driver of leukemogenesis, therapeutic resistance, and impaired myeloid differentiation [16]. The aim of this study was to comprehensively characterize *BCLAF1* mRNA isoforms and perform an in-depth analysis of the expression of transcriptional variants in AML patients compared to healthy controls using the GEPIA2 online tool. We found that the FL isoform was significantly upregulated compared to the healthy counterpart (*p* < 0.05), whereas the SL isoform did not show any statistically significant difference in expression (Fig. 1A). Similarly, in three leukemia cell lines (U937, K562, NB4), we observed an overexpression of the FL isoform compared to the SL isoform under basal conditions (Fig. 1B), with this imbalance particularly pronounced in the AML-derived U937 cell line. Consistent results were obtained in our cohort of 36 AML patient’s primary samples (Fig. 1C), whereas CD34^+^ human progenitor cells exhibited balanced expression of the two isoforms (Fig. 1C). The identity of these isoforms was further validated by sanger sequencing (Supplementary Fig. S1A). No significant differences in the expression of exon 11 isoforms were detected in any of the analyzed samples (Supplementary Fig. S1B).

The imbalance of *BCLAF1* FL and SL expression isoforms suggested their potential functional roles and regulatory mechanisms in AML. Our previous work demonstrated that selective epigenetic drugs, such as vorinostat (SAHA), a pan-HDAC inhibitor, is able to reverse the AML phenotype by negatively regulating the BCLAF1 FL isoform [16]. Since the FL isoform is critically involved in AML tumorigenesis, we examined BCLAF1 isoform regulation after SAHA (5 μM) treatment for 6, 24, and 48 hours. In all cell lines examined, SAHA modulated BCLAF1 isoform splicing, particularly after 24 h of treatment, at both RNA and protein level (Fig. 1D and Supplementary Fig. S1C). These findings were further confirmed by RNA-scope analysis (Supplementary Fig. S1D). In addition, we verified that the SL isoform mRNA detected after SAHA treatment was newly synthesized, highlighting the specific effect of SAHA on BCLAF1 splicing regulation (Fig. 1E). No significant differences were observed in the expression of isoforms differing by the presence or absence of exon 11 (Supplementary Fig. S1E).

We next evaluated a panel of inhibitors targeting key enzymes that are involved in the modulation of alternative splicing through epigenetic mechanisms, including HDACs, DNMTs, and Sirtuins to investigate epigenetic regulation of BCLAf1 isoforms. Modulation of *BCLAF1* splicing was observed exclusively following treatment with specific compounds (SAHA, MS-275, 5-azacytidine), resulting in a decrease in FL isoform expression accompanied by an increase in the SL isoform (Supplementary Fig. S1F-G). DNMT3A appears to play a central role in splicing mechanism, as treatment with the DNMT1/3A inhibitor SGI-1027 led to a reduction in FL isoform levels while stabilizing the SL isoform, whereas selective inhibition of DNMT1 with GSK3685032 produced opposite effects, allowing to potentially exclude DNMT1 involvement in *BCLAF1* splicing modulation (Fig. 1F). Similarly, based on the use of specific inhibitors, we hypothesize that HDAC1 may contribute to this splicing regulation mechanism (Supplementary Fig. S1H). MRG15also appears to participate in this process, as its inhibition increased FL isoform expression without affecting the SL isoform (Fig. 1F and Supplementary Fig. S1I). Lastly, since SRSF10 is known to be a regulator of BCLAF1 splicing [17], we assessed its expression following treatment with the tested compounds and found that a significant reduction occurred only after SAHA treatment, suggesting that SRSF10 is unlikely to mediate the observed modulation of BCLAF1 splicing (Supplementary Fig. 1J).

Our results demonstrate a marked dysregulation of BCLAF1 isoforms in AML, with significant upregulation of the full-length (FL) isoform over the short-length (SL) variant in both patient samples and leukemia cell lines. This imbalance suggests a key role for the FL isoform in AML pathogenesis. Notably, expression of another BCLAF1 isoform, differing by exon 11 inclusion, remained unchanged, emphasizing the specific involvement of the FL variant. In contrast, normal CD34+ progenitors showed balanced isoform expression, indicating a leukemia-specific alteration. These findings support the potential of the FL isoform as a diagnostic biomarker and therapeutic target. Additionally, the regulation of BCLAF1 splicing appears epigenetically controlled, involving DNMT3A, HDAC1, and MRG15. This reveals a coordinated mechanism contributing to leukemogenesis and suggests that targeting epigenetic modulators to restore isoform balance could be a novel therapeutic strategy.

### Identification and characterization of alternative splicing mechanisms regulating BCLAF1

**Fig. 2:**
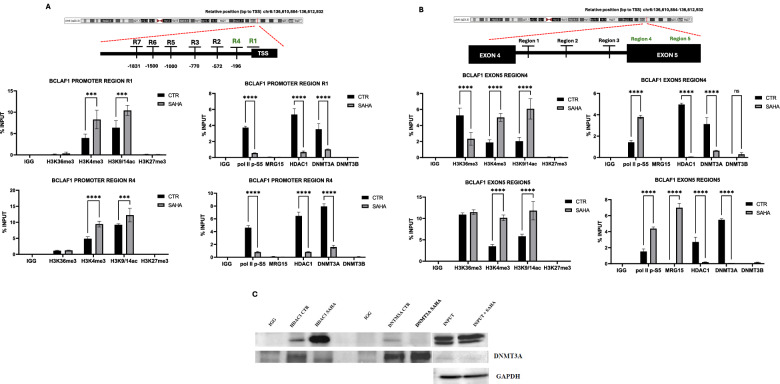
Epigenetic regulation of BCLAF1 promoter and exon 5 following SAHA treatment. **A** Chromatin immunoprecipitation (ChIP) analysis performed in U937 cells treated with SAHA (5 µM) for 24 h on BCLAF1 promoter region against HDAC1, DNMT3A, DNMT3B, MRG15, and RNA Polymerase II (Ser5P), and histone modifications H3K36me3, H3K4me3, H3K27me3, and H3K9/14ac. **B** ChIP analysis performed in U937 cells treated with SAHA (5 µM) for 24 h on BCLAF1 exon 5 region against HDAC1, DNMT3A, DNMT3B, MRG15, and RNA Polymerase II (Ser5P), and histone modifications H3K36me3, H3K4me3, H3K27me3, and H3K9/14ac. C Co-immunoprecipitation (Co-IP) analysis of HDAC1 and DNMT3A in U937 cells treated with SAHA for 24 h. Values are presented as the mean ± SEM. Significance was defined as a *p*-value < 0.05, indicated by asterisks in the figures (* = *p* < 0.05, ** = *p* < 0.01, *** = *p* < 0.001, **** = *p* < 0.0001).

BCLAF1 splicing is likely controlled by epigenetic mechanisms involving DNMT3A, HDAC1, and MRG15 as key regulators. To investigate chromatin modifications associated with BCLAF1 splicing regulation, we performed ChIP-qPCR analysis following SAHA treatment. Our analysis focused on the BCLAF1 promoter region (2000 bp upstream of the transcription start site) and the entire exon 5, including the adjacent intron 4. We examined histone modifications known to be involved in splicing regulation (H3K9/14ac, H3K36me3, H3K4me3, and H3K27me3) alongside chromatin-associated factors including MRG15, HDAC1, DNMT3A, DNMT3B, and RNA polymerase II phosphorylated at serine 5. As shown in Fig. 2A, promoter regions potentially involved in the regulation of BCLAF1 splicing are located within 600 bp upstream of the transcription start site and are denoted as R1 and R4 (additional regions are shown in Supplementary Fig. S2A). Following treatment with the HDAC inhibitor SAHA, we observed a pronounced increase in global histone acetylation (H3K9/14ac), consistent with the expected effect of HDAC inhibition, and accompanied by a significant enrichment of the active chromatin mark H3K4me3 at both R1 and R4. In contrast, the levels of H3K36me3 and H3K27me3 remained unchanged in these regions, indicating a selective effect on specific histone acetylation. In parallel, following SAHA treatment, we detected a marked reduction in the binding of DNMT3A and HDAC1 at both R1 and R4. This observation is consistent with the known inverse relationship between H3K4me3 enrichment and DNMT3A recruitment, suggesting that SAHA-mediated epigenetic changes may disrupt repressive chromatin complexes at the BCLAF1 promoter.

To validate the direct interaction between DNMT3A and HDAC1, we performed Co-IP assays. DNMT3A and HDAC1 strongly interacted under basal (untreated) conditions; notably, this interaction was completely disrupted following SAHA treatment (Fig. 2C). In line with our hypothesis, MRG15 binding was absent at these promoter regions, confirming that these sites are not directly involved in splicing-related exonic regulation. Importantly, DNMT3B binding remained unchanged, highlighting the specificity of the DNMT3A–HDAC1 interaction and its modulation by SAHA.

Focusing on the regions encompassing exon 5 and intron 4, significant changes were detected exclusively within exon 5, specifically in regions denoted as R4 and R5 (Fig. 2B and Supplementary Fig. 2B). In terms of histone modifications, SAHA treatment induced an increase in histone acetylation and H3K4me3 levels in R4, accompanied by a decrease in H3K36me3. In R5, we observed a similar increase in acetylation and H3K4me3, while H3K36me3 levels remained unchanged; H3K27me3 levels stayed constant in both regions.

An analysis of transcription factor dynamics revealed that RNA polymerase II binding increased significantly at both R4 and R5 following SAHA treatment. Notably, MRG15 binding increased significantly only at R5, coinciding with a reduction in DNMT3A and HDAC1 occupancy in both regions. Similarly to what we observed at the promoter regions, DNMT3B binding remained unchanged.

Our findings reveal that BCLAF1 splicing is epigenetically regulated by a complex involving DNMT3A and HDAC1, which binds its promoter under basal conditions. SAHA treatment alters chromatin histone marks, reducing DNMT3A/HDAC1 binding and increasing MRG15 recruitment to exon 5, promoting SL isoform expression. The absence of DNMT3B involvement and unchanged H3K36me3/H3K27me3 levels highlight the specificity of this regulatory mechanism.

### Dynamic interplay between DNMT3A and DNMT3B in regulating BCLAF1 alternative splicing

**Fig. 3:**
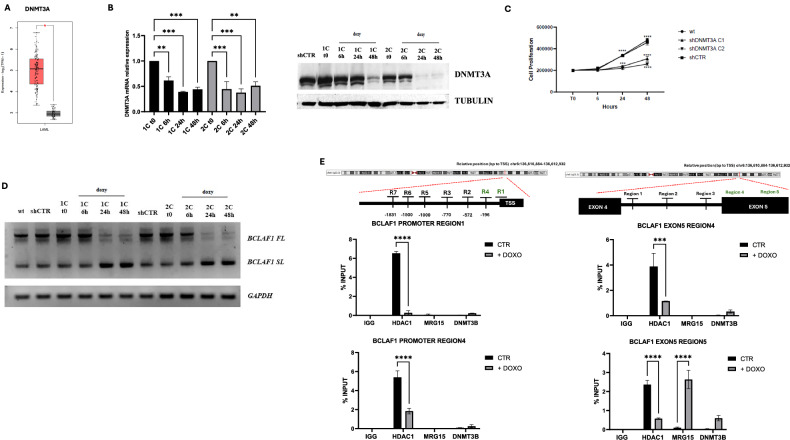
Analysis of BCLAF1 expression and epigenetic changes in DNMT3A knockout U937 clones. **A** GEPIA analysis on DNMT3A expression in AML patient samples. **B** Real-time PCR and Western blot analyses of DNMT3A expression in U937 sh-DNMT3A clones after doxycycline induction (2 µg/mL) at 6, 24, and 48 h. **C** Cell proliferation assay by cell counting in U937 sh-DNMT3A clones after doxycycline induction (2 µg/mL) at 6, 24, and 48 h. **D** PCR analysis of BCLAF1 expression in U937 sh-DNMT3A clones after doxycycline induction (2 µg/mL) at 6, 24, and 48 h. **E** ChIP analysis performed in U937 sh-DNMT3A clones after 48 h of doxycycline induction (2 µg/mL) on BCLAF1 promoter and exon 5 regions against HDAC1, DNMT3A, DNMT3B, MRG15, and RNA Polymerase II (Ser5P), and histone modifications H3K36me3, H3K4me3, H3K27me3, and H3K9/14ac. Values are presented as the mean ± SEM. Significance was defined as a *p*-value < 0.05, indicated by asterisks in the figures (* = *p* < 0.05, ** = *p* < 0.01, *** = *p* < 0.001, **** = *p* < 0.0001).

In AML, BCLAF1 FL isoform overexpression likely arises from aberrant DNMT3A/HDAC1 activity and reduced MRG15-H3K36me3 binding, impairing PTB recruitment and splicing regulation. Using the GEPIA2 tool, we identified a significant overexpression of DNMT3A in AML samples compared to healthy controls (Fig. 3A). To investigate the specific role of DNMT3A, we generated two inducible U937 cell clones with DNMT3A knockdown (Fig. 3B). As shown in Fig. 3C, DNMT3A silencing led to a decrease in cell proliferation, correlating with reduced *BCLAF1* expression [16]. Importantly, no changes were observed in the expression of DNMT3B, DNMT1, SRSF10, or HDAC1 (Supplementary Fig. 3A, B). Subsequently, lentivirus-mediated knockdown of DNMT3A in U937 induced a specific modulation of BCLAF1 splicing, corroborating the role of DNMT3A in regulating the production of its isoforms (Fig. 3D). Specifically, DNMT3A silencing shifted the balance between FL and SL isoforms, favoring increased production of the latter.

To assess whether DNMT3A depletion affects the recruitment of chromatin factors, we performed ChIP-qPCR after 48 h of doxycycline induction. DNMT3A knockdown caused a significant reduction in HDAC1 binding at the promoter regions previously analyzed (Fig. 3E). Similarly, HDAC1 binding decreased at exon 5, accompanied by a notable increase in MRG15 occupancy. No significant changes were observed in DNMT3B binding.

**Fig. 4:**
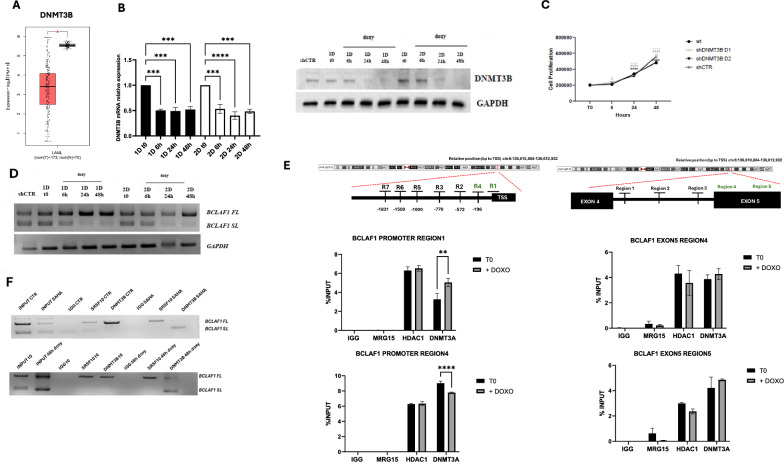
Analysis of BCLAF1 expression and epigenetic changes in DNMT3B knockout U937 clones. **A** GEPIA analysis on DNMT3B expression in AML patient samples. **B** Real-time PCR and Western blot analyses of DNMT3B expression in U937 sh-DNMT3A clones after doxycycline induction (2 µg/mL) at 6, 24, and 48 h. **C** Cell proliferation assay by cell counting in U937 sh-DNMT3B clones after doxycycline induction (2 µg/mL) at 6, 24, and 48 h. **D** PCR analysis of BCLAF1 expression in U937 sh-DNMT3B clones after doxycycline induction (2 µg/mL) at 6, 24, and 48 h. **E** ChIP analysis performed in U937 sh-DNMT3B clones after 48 h of doxycycline induction (2 µg/mL) on BCLAF1 promoter and exon 5 regions against HDAC1, DNMT3A, DNMT3B, MRG15, and RNA Polymerase II (Ser5P), and histone modifications H3K36me3, H3K4me3, H3K27me3, and H3K9/14ac. **F** RNA immunoprecipitation assays performed in U937 cells treated with SAHA (5 µM) for 24 h and in U937 sh-DNMT3A knockout clones after 48 h of doxycycline (2 µg/mL) induction for DNMT3B on BCLAF1 RNA. Values are presented as the mean ± SEM. Significance was defined as a *p*-value < 0.05, indicated by asterisks in the figures (* = *p* < 0.05, ** = *p* < 0.01, *** = *p* < 0.001, **** = *p* < 0.0001).

Conversely, GEPIA2 analysis revealed a significant downregulation of DNMT3B in AML samples compared to healthy controls (Fig. 4A). Given the potential compensatory relationship between DNMT3A and DNMT3B, we generated two inducible U937 clones with DNMT3B knockdown (Fig. 4B). Silencing DNMT3B did not significantly affect proliferation in these cells (Fig. 4C). Furthermore, expression levels of DNMT3A, DNMT1, SRSF10, and HDAC1 remained unchanged upon DNMT3B knockdown (Supplementary Fig. S4A-B). Surprisingly, DNMT3B depletion shifted the balance of *BCLAF1* isoforms, promoting an increase in the FL isoform (Fig. 4D). To assess whether DNMT3B loss affects the binding of DNMT3A, HDAC1, and MRG15, we performed ChIP-qPCR in the DNMT3B knockdown clones. DNMT3B depletion appeared to stabilize or enhance the basal binding of these factors (Fig. 4E).

To explore how DNMT3B regulates BCLAF1 alternative splicing, we investigated interaction between MRG15 and DNMT3B using Co-IP assays. No interaction between these proteins was detected (Supplementary Fig. S4C). Considering reports that DNMT3B may also function as an RNA-binding protein [18, 19], we next examined whether DNMT3B binds BCLAF1 RNA following SAHA treatment and also in DNMT3A-depleted U937 clones. We observed that DNMT3B bound the *BCLAF1* FL RNA under basal conditions; notably, after SAHA treatment and DNMT3A depletion, DNMT3B also bound the SL RNA isoform (Fig. 4F).

Our results show that DNMT3A and DNMT3B regulate BCLAF1 splicing in AML via distinct mechanisms. DNMT3A, overexpressed in AML, promotes the FL isoform by recruiting HDAC1 and blocking MRG15 binding. Its knockdown reduces proliferation and shifts splicing toward the SL isoform. Conversely, DNMT3B is downregulated and normally suppresses the FL isoform. It regulates splicing by directly binding BCLAF1 RNA, not via DNA methylation, and acts independently of MRG15. These findings suggest DNMT3A and DNMT3B control BCLAF1 splicing through separate but complementary pathways.

### Influence of epigenetics on BCLAF1 post-translational modification (PTM) landscape

**Fig. 5:**
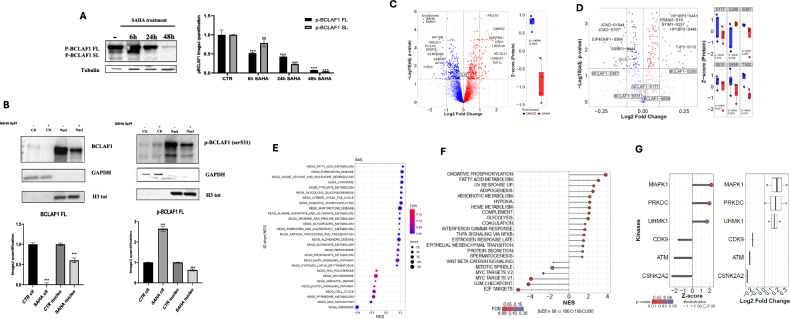
Proteomic analysis in U937 cells treated with SAHA. **A** Western blot analysis on BCLAF1 phosphorylation at Ser531 in U937 cells after treatment with SAHA (5 µM) at 6, 24, and 48 h. **B** Nucleus/cytosol Western blot analysis of phosphorylated BCLAF1 (Ser531) after 24 h of SAHA treatment (5 µM). **C** Mass spectrometry-based proteomic analysis of U937 cells treated with SAHA (5 µM) at 24 h. **D** Mass spectrometry-based phosphoproteomic analysis of U937 cells treated with SAHA (5 µM) at 24 h. Values are presented as the mean ± SEM. Significance was defined as a *p*-value < 0.05, indicated by asterisks in the figures (* = *p* < 0.05, ** = *p* < 0.01, *** = *p* < 0.001, **** = *p* < 0.0001). **E** KEGG pathway analysis of the MS dataset of U937 cells treated with SAHA (5 µM) at 24 h (FDR q-value < 0.05). **F** Normalized enrichment score (NES) of Hallmark pathways of the MS dataset of U937 cells treated with SAHA (5 µM) at 24 h (FDR q value < 0.05). **G** Kinase-Substrate Enrichment Analysis (KSEA; v0.99.0) of the phosphoproteomic data of U937 cells treated with SAHA (5 µM) at 24 h (KIN Cutoff ( > 5).

BCLAF1 is able to shuttle between the nucleus and cytosol via an HDAC4-dependent pathway, and its differential localization appears to be linked to its functional roles [16]. We focused on a specific PTM of BCLAF1, phosphorylation at serine 531, given that the nuclear export signal sequences of BCLAF1 are located near exon 5a, close to serine 531 [20], (Supplementary Fig. S5A). This phosphorylation is also reported to be involved in cancer progression [21]. Following SAHA treatment, we observed an increase in phosphorylation detectable in both the FL isoform (up to 48 h) and the truncated isoform (up to 24 h) (Fig. 5A and Supplementary Fig. S5B). We also assessed BCLAF1 subcellular localization by performing nucleus/cytosol fractionation after 24 h of SAHA treatment to investigate potential changes in compartmentalization. Post-treatment, BCLAF1 expression was predominantly nuclear, although the FL isoform band was also faintly detectable in the cytosolic fraction (Fig. 5B). Phospho-BCLAF1 analysis revealed a predominantly nuclear localization; however, following 24 h of SAHA treatment, we detected an increased presence of the phosphorylated protein in the cytosolic compartment (Fig. 5B). This redistribution suggests PTMs, particularly phosphorylation, may modulate BCLAF1 subcellular localization. To further explore this possibility, we investigated the effects of other HDAC inhibitors; specifically, U937 cells were treated with MS-275, a class I-selective HDAC inhibitor. We observed a marked reduction in total BCLAF1 FL protein levels. Interestingly, in contrast to SAHA, MS-275 treatment resulted in a more prominent accumulation of BCLAF1 in the cytosolic fraction, as revealed by nuclear/cytosolic protein fractionation (Supplementary Fig. S5C).

To validate our observations, we performed MS-based proteomic and phosphoproteomic analyses comparing untreated U937 cells to those treated with SAHA for 24 h. Consistent with immunoblot data, global proteomic analysis revealed a significant reduction in total BCLAF1 protein levels following SAHA exposure (Fig. 5C). In contrast, phosphoproteomic profiling uncovered distinct and dynamic changes in phosphorylation at specific residues: phosphorylation at serine 531, which we previously associated with nuclear export regulation, was reduced, whereas phosphorylation at serine 290 was significantly increased (Fig. 5D). KEGG analysis of the MS dataset showed that the following pathways are significantly downregulated: ribosome biogenesis, DNA replication, pyrimidine metabolism, cell-cycle control, mismatch repair, spliceosome function, and Notch signaling (Fig. 5E). Notably, suppression of the spliceosome pathway aligns with BCLAF1’s role in RNA processing, providing a mechanistic basis for the reduced expression of splicing-related genes. Consistently, Gene Set Enrichment Analysis (GSEA) of the global proteomic profile (Fig. 5F) and Kinase-Substrate Enrichment Analysis (KSEA) of phosphoproteomic data (Fig. 5G) revealed downregulation of proliferation-associated gene sets and key cell-cycle kinases, including CDK9, ATM, and CSNK2A2.

Our findings show that BCLAF1 is regulated by post-translational modifications, especially phosphorylation, in response to HDAC inhibitors. SAHA treatment increases phosphorylation at serine 531 in both FL and truncated isoforms, suggesting a role in protein stability, activity, or nuclear export due to its proximity to the export signal. Differences in BCLAF1 localization after SAHA vs. MS-275 support this. Phosphoproteomics revealed reduced serine 531 phosphorylation at later time points, hinting at turnover or altered interactions, and increased phosphorylation at serine 290, potentially reflecting alternative or compensatory regulation. These events likely fine-tune BCLAF1 function and localization.

## Discussion

In our study, we discovered distinct BCLAF1 isoforms in AML and proved that their expression can be modulated by treatment with epigenetic drugs. Specifically, these treatments led to a reduction in the FL isoform and a concomitant increase in the SL isoform, indicating a drug-induced shift in BCLAF1 alternative splicing. Our findings identify the distinct yet complementary roles of DNMT3A and DNMT3B in regulating this splicing event. DNMT3A modulates BCLAF1 splicing through chromatin-associated mechanisms, promoting HDAC1 recruitment and inhibiting MRG15 binding, thereby favoring the expression of the FL isoform. Upon DNMT3A depletion, this regulatory balance is disrupted, leading to enhanced MRG15 binding and increased SL isoform production. In contrast, DNMT3B appears to act antagonistically to DNMT3A. DNMT3B knockdown results in elevated expression of the FL isoform, suggesting a repressive role under basal conditions. Notably, our data demonstrate that DNMT3B binds directly to BCLAF1 RNA (preferentially to the FL isoform under basal conditions, and to the SL isoform following DNMT3A depletion or SAHA treatment), indicating that DNMT3B influences splicing via an RNA-mediated mechanism, possibly independently of its canonical role in DNA methylation. While this work provides novel insights into the epigenetic regulation of BCLAF1 splicing in AML, some questions rise. It remains to be established the precise mechanisms by which DNMT3A and DNMT3B coordinate the recruitment of splicing factors and the involvement of additional chromatin modifiers. Is the DNMT-dependent splicing control of BCLAF1 a general mechanism, possibly extendable to other families or factors? It is tempting to speculate that DNMT3A deregulation in AML (and in other cancer types) might drive splicing aberrations linking epigenetics and chromatin deregulation to RNA functions. The fact that DNMT3A may coordinate splicing mechanisms also binds this deregulation to the possible maturation block and stem state [22]. It is tempting to speculate that BCLAF1 splicing re-instatement via epidrugs resetting might turn helpful as potential differentiation or anti leukemic therapy. Also, the potential impact of the frequently occurring DNMT3A mutations in AML on these mechanisms stays to be defined and might add further knowledge.

Overall, our findings underscore the critical role of DNMT3A in splicing regulation through chromatin remodeling and reveal a previously unrecognized function for DNMT3B in modulating splicing via RNA binding. These insights not only deepen our understanding of epigenetic control of splicing in AML but also suggest new potential targets for therapeutic intervention.

## Supplementary information

Supplementary Figures

Supplementary Methods

Supplementary Information

Data Set 2

## Data Availability

The mass spectrometry proteomics data have been deposited to the ProteomeXchange Consortium via the PRIDE partner repository with the dataset identifier PXD063064. Full-length, uncropped images of all Western blots and gels used in this study are available in the Supplementary Information.
