# Supplementary material for: Dynamic epigenetic regulation of BCLAF1 splicing in acute myeloid leukemia
Source: Cell Death Dis. 2026 Mar 24;17(1):344. doi: 10.1038/s41419-026-08594-4 (PMC13039510; doi:10.1038/s41419-026-08594-4)
Supplement: Supplementary file 2 — Supplementary Methods [file 41419_2026_8594_MOESM2_ESM.docx]

**Supplementary Methods**

**Reagents**

Chemical compounds used in this study and their corresponding concentrations are listed in Table 1; all compounds were dissolved in DMSO (Sigma-Aldrich). Primer sequences used for PCR and RT-PCR are listed in Table 2, antibodies in Table 3, and details of kits in Table 4.

| **Compound** | **Function** | **Concentration** |
| --- | --- | --- |
| **Vorinostat (SAHA)**  **(Merck, Steinheim, Germany)** | Pan-HDAC inhibitor | 5 μM |
| **Entinostat (MS-275)**  **(Alexis Biochemicals, Rome, Italy)** | Class I HDAC inhibitor | 5 μM |
| **5-Azacytidine**  **(Merck, Steinheim, Germany)** | DNMT inhibitor | 5 μM |
| **F2F-2020178-00X**  **(kindly provided by Prof. Summa, University of Naples “Federico II”, Italy)** | HDAC6-specific inhibitor | 5 μM |
| **Trichostatin A**  **(Merck, Steinheim, Germany)** | Pan-HDAC inhibitor | 5 μM |
| **EX-527**  **(Merck, Steinheim, Germany)** | SIRT1 inhibitor | 5 μM |
| **Meclofenamic acid**  **(Merck, Steinheim, Germany)** | FTO inhibitor | 5 μM |
| **Cisplatin**  **(Merck, Steinheim, Germany)** | DNA damaging agent | 5 μM |
| **Tasquinimod**  **(Merck, Steinheim, Germany)** | HDAC4 inhibitor | 5 and 50 μM |
| **Santacruzamate A**  **(Selleckchem, Zürich, Switzerland)** | HDAC2 inhibitor | 5, 10, and 50 μM |
| **EML 951**  **(kindly provided by Prof. Sbardella, University of Salerno, Italy)** | Novel inhibitor of the methyl-lysine reader protein MRG15 | 5, 10, and 50 μM |
| **GSK3685032**  **(Selleckchem, Zürich, Switzerland)** | DNMT1 inhibitor | 5 and 50 μM |
| **SGI1027**  **(Selleckchem, Zürich, Switzerland)** | DNMT1/3A inhibitor | 5 and 50 μM |
| **Doxocyclin**  **(Selleckchem, Zürich, Switzerland)** | Antibiotic | 2 µg/mL |

**Table 1**: List of chemical compounds

| **Primer Sequences** | |
| --- | --- |
| **BCLAF1 mRNA FW** | TTCGATCCATCTTTGACCACA |
| **BCLAF1 mRNA RW** | TGATACGAAGTGAACCGCTCG |
| **BCLAF1(-5a) splicing FW** | TGGGTCTGGTTCTGTTGGAAAT |
| **BCLAF1(-5a) splicing RW** | AGCAAGCAGCCTGTCTTTAGTC |
| **GAPDH FW** | GGAGTCAACGGATTTGGTCGT |
| **GAPDH RW** | GCTTCCCGTTCTCAGCCTTGA |
| **SRSF10 FW** | CCCCCAACACGTCTCTGTTC |
| **SRSF10 RW** | GGACGGCGAGTGTAGAAATCA |
| **GAPDH promoter region FW** | CAATTCCCCATCTCAGTCGT |
| **GAPDH promoter region RW** | GCAGCAGGACACTAGGGAGT |
| **BCLAF1 promoter regions prom1 FW** | GCTTGCCCGCATATTTAG |
| **BCLAF1 promoter regions prom1 RW** | TTTCCCGACTCAAGAACG |
| **BCLAF1 promoter regions prom2 FW** | GATCAGACGCCACGAAAGGG |
| **BCLAF1 promoter regions prom2 RW** | CAAGACGTGGCTGTAATTGC |
| **BCLAF1 promoter regions prom3 FW** | TACGTTGGGCTGTGTTCCTC |
| **BCLAF1 promoter regions prom3 RW** | CGGCGTGATGACAGCTTTAC |
| **BCLAF1 promoter regions prom4 FW** | GCAATTACAGCCACGTCTTG |
| **BCLAF1 promoter regions prom4 RW** | GTAGAGTAGGGTGGGTGTTTTG |
| **BCLAF1 promoter regions prom5 FW** | CAAAACACCCACCCTACTCTAC |
| **BCLAF1 promoter regions prom5 RW** | CTAAATATGCGGGCAAGC |
| **BCLAF1 promoter regions prom6 FW** | AGGCTTTGTTCCAAAGGATCTC |
| **BCLAF1 promoter regions prom6 RW** | CGCTCCCAATACATGCTGTT |
| **BCLAF1 promoter regions prom7 FW** | TCTCAGCATTCTCTTTGTACCTT |
| **BCLAF1 promoter regions prom7 RW** | AAACGCCGAAAAGAGCAGAG |
| **BCLAF1 exon5 region1 FW** | CTGGTGAACTTTGGAAGCTAAGTG |
| **BCLAF1 exon5 region1 RW** | GCTCTAAGCTACTCAACTTCTCC |
| **BCLAF1 exon5 region2 FW** | GGAATAGCACTGCACAATC |
| **BCLAF1 exon5 region2 RW** | CTAATAGATAATACATTG |
| **BCLAF1 exon5 region3 FW** | GGCAATCTCTAGAAGTTCGTGTTAG |
| **BCLAF1 exon5 region3 RW** | GTTGCCCAATCCCTCAGGACC |
| **BCLAF1 exon5 region4 FW** | GAGGCTTCAAAAGAGAAAGG |
| **BCLAF1 exon5 region4 RW** | GGACTTGTACTTGAGTCC |
| **BCLAF1 exon5 region5 FW** | GAGACTGGATATGTAGTGGAAAGG |
| **BCLAF1 exon5 region5 RW** | TCATTTTGATCCTAAGTGG |
| **HDAC1 FW** | GACAAGGCCACCCAATGAAG |
| **HDAC1 RW** | GCTTGCTGTACTCCGACATG |
| **DNMT3A FW** | TGCAATGACCTCTCCATCGT |
| **DNMT3A RW** | CTTGTCACTAACGCCCATGG |
| **DNMT3B FW** | CCATGAAGGTTGGCGACAA |
| **DNMT3B RW** | TGGCATCAATCATCACTGGATT |
| **DNMT1 FW** | CGGTTCTTCCTCCTGGAGAATGTCA |
| **DNMT1 RW** | CACTGATAGCCCATGCGGACCA |

**Table 2.** Primer sequences used for PCR and RT-PCR

| **Antibody** | **Manufacturer** | **Catalog number** |
| --- | --- | --- |
| **Anti-BTF** | Bethyl Laboratories | A300-610A |
| **Anti-BCLAF1** | Invitrogen | PA5-52320 |
| **Anti-SRSF10** | Atlas Antibodies | HPA053831 |
| **Phospho-BCLAF1** | Invitrogen | PA5-105970 |
| **Anti-GAPDH** | Elabscience | E-AB-40337 |
| **HDAC1** | Diagenode | C15410325 |
| **DNMT3A** | Invitrogen | 64B1446 |
| **DNMT3B** | Invitrogen | PA1-884 |
| **Anti-Alpha tubulin** | Elabscience | E-AB-20036 |
| **H3K9/14ac** | Diagenode | pAb-005-050 |
| **MORF4L1/MRG15** | Cell Signaling | 14098 |
| **H3K4me3** | Diagenode | C15310003 |
| **H3K36me3** | Cell Signaling | 4909 |
| **H3K27me3** | Diagenode | C15410195 |
| **RNA polymerase II CTD repeat YSPTSPS** | Abcam | Ab5408 |

**Table 3.** List of antibodies

| **Kit** | **Manufacturer** | **Catalog number** |
| --- | --- | --- |
| **Click-iT™ Nascent RNA Capture Kit** | ThermoFisher Scientific | C10365 |
| **RNAscope Multiplex Fluorescent Reagent Kit v2** | Bio-Techne | 323110, ACD |
| **EZ-Magna RIP RNA-Binding Protein Immunoprecipitation Kit** | Merck | 17-701 |

**Table 4.** List of kits

**Protein extraction and Western blot**

Cells were lysed in RIPA buffer (1 M Tris-HCl, 2 M NaCl, 1% NP-40, 10% SDS, 0.5 M EDTA) supplemented with 0.1 M Na_3_VO_4_, and Protein Inhibitor Cocktail (Applied Biological Materials). Electrophoresis was carried out in polyacrylamide gel, and electroblotting on nitrocellulose membrane. Nuclear/cytosolic extraction was performed as described in [18]. All antibodies were used according to the manufacturer’s instructions. Immunoreactive signals were detected with a horseradish peroxidase-conjugated secondary antibody (Cytiva, Healthcare Amersham). Experiments were repeated at least three times.

**Co-immunoprecipitation (Co-IP)**

Co-IP was performed using whole cell lysate (1500 µg) in Co-IP buffer (150 mM NaCl, 50 mM Tris pH 7.0, 5% glycerol, 5 mM EDTA, 0.15% 185 Nonidet P-40, 1 mM DTT, 0.2 mM PMSF, 1 mM Na_3_VO_4_, and 1X Roche Protease Inhibitor Cocktail Tablets) as described in [16].

**Real-time PCR, PCR, and gel extraction**

Gene expression was evaluated by qRT-PCR; cDNA (50 ng) was amplified after using Power SYBR® Green PCR Master Mix 1X (Applied Biosystems). Normalization was obtained using the “housekeeping” gene GAPDH. The analysis was conducted using the ΔΔCt method and plotted with GraphPad Prism 8. PCR using selected primers for BCLAF1 FL and SL isoforms was performed using Taq 2X Master Mix from DreamTaq DNA Polymerase (ThermoFisher Scientific), and the products were run on 2% agarose gel. A total of 25 μL of the PCR reaction was run on a 2% agarose gel and amplicons were extracted using a QIAquick Gel Extraction Kit (Qiagen). Briefly, after recovering the band of interest from the gel in a 1.5 mL tube, three Buffer QG solution volumes were added. Samples were Sanger sequenced by Bio-Fab Research using an Applied Biosystems ABI PRISM 3730 capillary electrophoretic run sequencer.

**Chromatin immunoprecipitation followed by quantitative (ChIP-qPCR)**

U937 cells were cultured in RPMI supplemented with 10% FBS at 37°C and treated for 24 h with 5 µM SAHA. Chromatin was harvested as described in [20]. ChIP experiments were performed using H3K9/14ac (Diagenode, pAb-005-050), H3K4me3 (Diagenode, C15310003), H3K36me3 (Cell Signaling, 4909), and H3K27me3 (Diagenode, C15410195), RNA polymerase II CTD repeat YSPTSPS (Abcam, Ab5408), MORF4L1/MRG15 (Cell Signaling, 14098), HDAC1 (Diagenode, C15410325), DNMT3A (Invitrogen, 64B1446), and DNMT3B antibodies (Invitrogen, PA1-884) antibodies, followed by qPCR analysis. The primers used are listed in Table 2.

**Lentiviral vector production and cell transduction**

HEK293FT cells were transfected using Lipofectamine 3000 reagent (ThermoFisher Scientific) according to the manufacturer’s instructions. Lentiviral vector production was performed as previously described [19]. The lentiviral plasmid DNA used for DNMT3A and DNMT3B silencing was shERWOODUltramiR Lentiviral Inducible (Vector = pZIP TRE3G-ZsGreen-Puro, Transomic Technologies).

**RNAscope**

RNAscope was performed using an RNAscope Multiplex Fluorescent Detection Reagent Kit v2 (Advanced Cell Diagnostics, 323110). After washing in PBS 1X, the cells were fixed in PFA 4% overnight at 4°C. The day after, cells were washed in PBS 1X and then incubated with protease III. Before overnight incubation with the BCLAF1 probe at 40°C, cells were washed using MQ H_2_O. On the third day, the samples were first incubated 30 min with AMP-1 solution, and then 30 min with AMP-2 and 15 min with AMP-3 solutions. Subsequently, HPR-C1 and TSA fluorescein (1:1000) were used to incubate the cells for 15 min and 30 min, respectively. HPR blocker solution was used to incubate the cells for 15 min before setting up the slides using ProLong Antifade Mountant with DAPI (Invitrogen). All incubations on the third day were performed at 40°C.

**Proteomic and phosphoproteomic analysis**

Cells were lysed in RIPA buffer with protease inhibitors (Halt Protease Inhibitor and 5 mM EDTA; ThermoFisher Scientific), followed by sonication (20 cycles: 30 s ON, 30 s OFF) on a Bioruptor Plus instrument (Diagenode), and centrifuged at 14,000 × g for 20 min at 4°C. Protein supernatants were collected, and concentration was measured with the bicinchoninic acid assay (ThermoFisher Scientific, A65453), according to the manufacturer’s instructions. A total of 100 µg of protein lysate (per sample) was precipitated in ice-cold (−20ºC) methanol, resuspended in 7 M urea in 100 mM ammonium bicarbonate buffer (AMBIC), and reduced by adding tris(2-carboxyethyl)phosphine in 100 mM AMBIC and then incubating for 1 h at 37°C. Alkylation was performed by adding iodoacetamide in AMBIC and incubating for 30 min in the dark at room temperature. Urea was then diluted to 1 M with AMBIC, and Trypsin/Lys-C (Promega, V5071) was added in a 1:100 W/W ratio (enzyme/protein) to the samples, which were incubated at 37°C for 16 h under agitation. Formic acid was then added to acidify the peptide mixtures. Samples were centrifuged at 14,000 × g for 20 min at 4°C, supernatants were collected, and peptide mixtures were desalted using peptide desalting spin columns (ThermoFisher Scientific) according to the manufacturer’s instructions. Eluates were dried in a SpeedVac, resuspended in triethylammonium bicarbonate, and peptides were chemically labelled using Tandem Mass Tag (TMT) 10plex (ThermoFisher Scientific) according to the manufacturer’s instructions. Labelled samples were then pooled and dried. The mass spectrometry proteomics data have been deposited to the ProteomeXchange Consortium via the PRIDE partner repository with the dataset identifier PXD063064"

**High-pH sample fractionation**

TMT10 pools were fractionated using a Waters XBridge BEH130 C18 Column (3.5 μm, 2.1 × 150 mm column) coupled to an Ultimate 3000 system (ThermoFisher Scientific). Equilibration and peptide elution buffers were composed of 20 mM of ammonium formate in high-grade water and 10 mM ammonium formate in 90% V/V acetonitrile (ACN), respectively. Gradient was run as follows (constant flow of 200 μL/min): 3 min at 0% B, 35% B in 94 min, 80% B in 1 min, and 10 min at 80% B. Fractions were collected for the first 97 minutes using a ThermoFisher Scientific fraction collector, for a total of 96 fractions. Of these, 90% of the eluted volume was concatenated into 12 fractions for downstream phosphopeptide enrichment, while the remainder was concatenated in 24 fractions for proteome analysis. Concatenation scheme for the 96-well plates containing eluates was as follows: fraction 1 (well A1, C1, E1, G1), fraction 2 (well A2, C2, E2, G2), etc. Concatenated fractions were dried and frozen until downstream processing or mass spectrometry (MS) analysis.

**Phosphopeptide enrichment**

Samples for spectral library preparation (cell line and AllPrep flowthrough fractions) and clinical cohort analysis (SCAN-B AllPrep flowthroughs) were processed according to a previously published automated phosphopeptide enrichment protocol 1 [21]. Briefly, peptide mixtures from high-pH fractionation or directly from C18 desalting were loaded on an AssayMAP Bravo platform. The Fe(III)-NTA cartridges used here were primed with 50% V/V ACN, 0.1% V/V trifluoroacetic acid, equilibrated with loading buffer (80% V/V ACN and 0.1% V/V trifluoroacetic acid in high-grade water). Peptide mixtures were then loaded on phosphoenrichment cartridges, washed with loading buffer, and eluted with 5% V/V ammonia solution into a 50% V/V FA solution (eluate plate). Samples were then dried and stored at −80°C until MS analysis.

**MS analysis and file processing**

TMT pools were analyzed on a Q Exactive HF-X system (ThermoFisher Scientific) coupled to a DionexUltiMate 3000 high-performance liquid chromatography system (ThermoFisher Scientific). Peptides were trapped on a μPAC Neo trap columns and separated on a μPA Neo 50 cm column; mobile phases and flow used in this analysis were the same as above. Peptides were eluted in a 140 min gradient (flow: 400 nL/min; mobile phase A: 0.1% formic acid in H_2_O; mobile phase B: 100% ACN and 0.1% formic acid). The chromatographic gradient was run as follows: 6% B for 3 min; 6–25% B in 110 min; 25–40% B in 10 min; 40–99% B in 1 min; 99% B for 10 min. The 15 most abundant peaks from the MS scan (resolution: 120,000 at 200 m/z) were selected and fragmented by higher energy induced collision dissociation (collision energy: 34). Dynamic exclusion was activated (window: 30 s) Automatic gain control target for full MS and MS/MS scans were set to 3E6 and 1E3, respectively. Thermo RAW files were processed using FragPipe (v22.0; <https://fragpipe.nesvilab.org/>) with the built-in TMT10 method. For analysis of the phosphoproteome files, the STY option was enabled in the modification tab of the MSFragger panel. A FASTA file used for DDA and DIA searches was downloaded from Uniprot ([www.uniprot.org](https://www.uniprot.org)), selecting only the Swissprot subset (v2024.01.15).

**Proteomic data analysis**

Proteome and phosphosite tables were filtered for missing data (transcriptome: 30%, proteome: 40%; phosphoproteome: 80%) and mean-centered across samples and features (genes, proteins, phosphosites). For proteomic and phosphoproteomic dataset tables, batch effect correction and quantile normalization scripts were performed with limma (v3.46.0) in R (v4.0.5). Pathway analyses were performed with Gene Set Enrichment Analysis (GSEA; v4.1.0) [22] using the Hallmarks and Reactome databases (from MSigdDB, v2024.01). GSEA settings were as follows: gene set permutation type, classic scoring method, t-test metric. False discovery rate cutoff for significant pathways was set to 0.25. Reactome pathways were plotted using the Enrichment Map plug-in in Cytoscape (v3.8.2). Sample-wise pathway scores were calculated using Gene Set Variation Analysis (GSVA; v1.38.2) [23] in R. Phosphoproteomic data were analyzed by kinase-substrate enrichment analysis (KSEA; v0.99.0) [24] in R. Here, Network KIN terms were allowed with a cutoff of 5. Enriched kinases were selected based on p-value (< 0.05) and number of substrates (> 2).

**Web sites/database referencing**

The web resources GEPIA2 (<http://gepia2.cancer-pku.cn/#index>) and BioRender (<https://www.biorender.com/>) were employed.
