# Supplementary material for: Dynamic epigenetic regulation of BCLAF1 splicing in acute myeloid leukemia
Source: Cell Death Dis. 2026 Mar 24;17(1):344. doi: 10.1038/s41419-026-08594-4 (PMC13039510; doi:10.1038/s41419-026-08594-4)
Supplement: Supplementary file 3 — Supplementary Information [file 41419_2026_8594_MOESM3_ESM.pdf]

FIGURE 1C

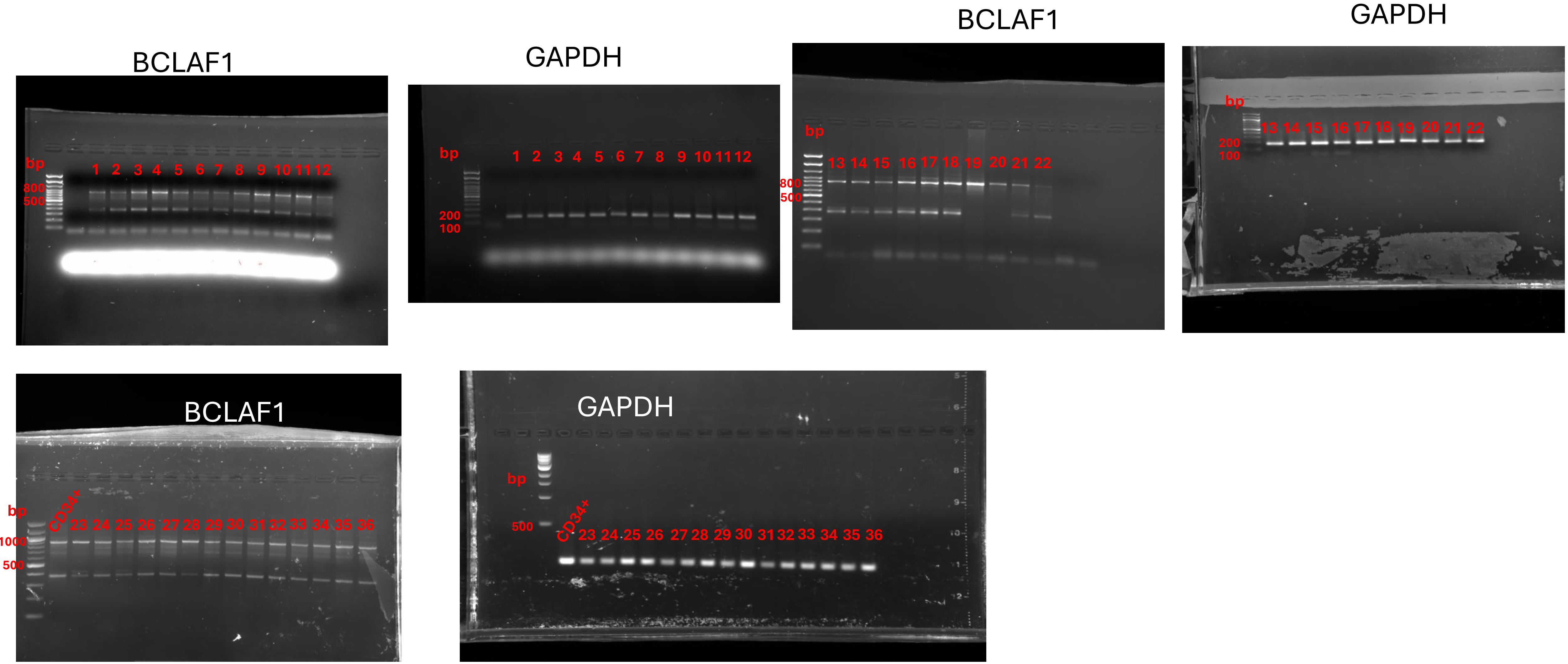

Figure 1C. PCR on AML patients for BCLAF1 and GAPDH #1-36 + CD34+

FIGURE 1D

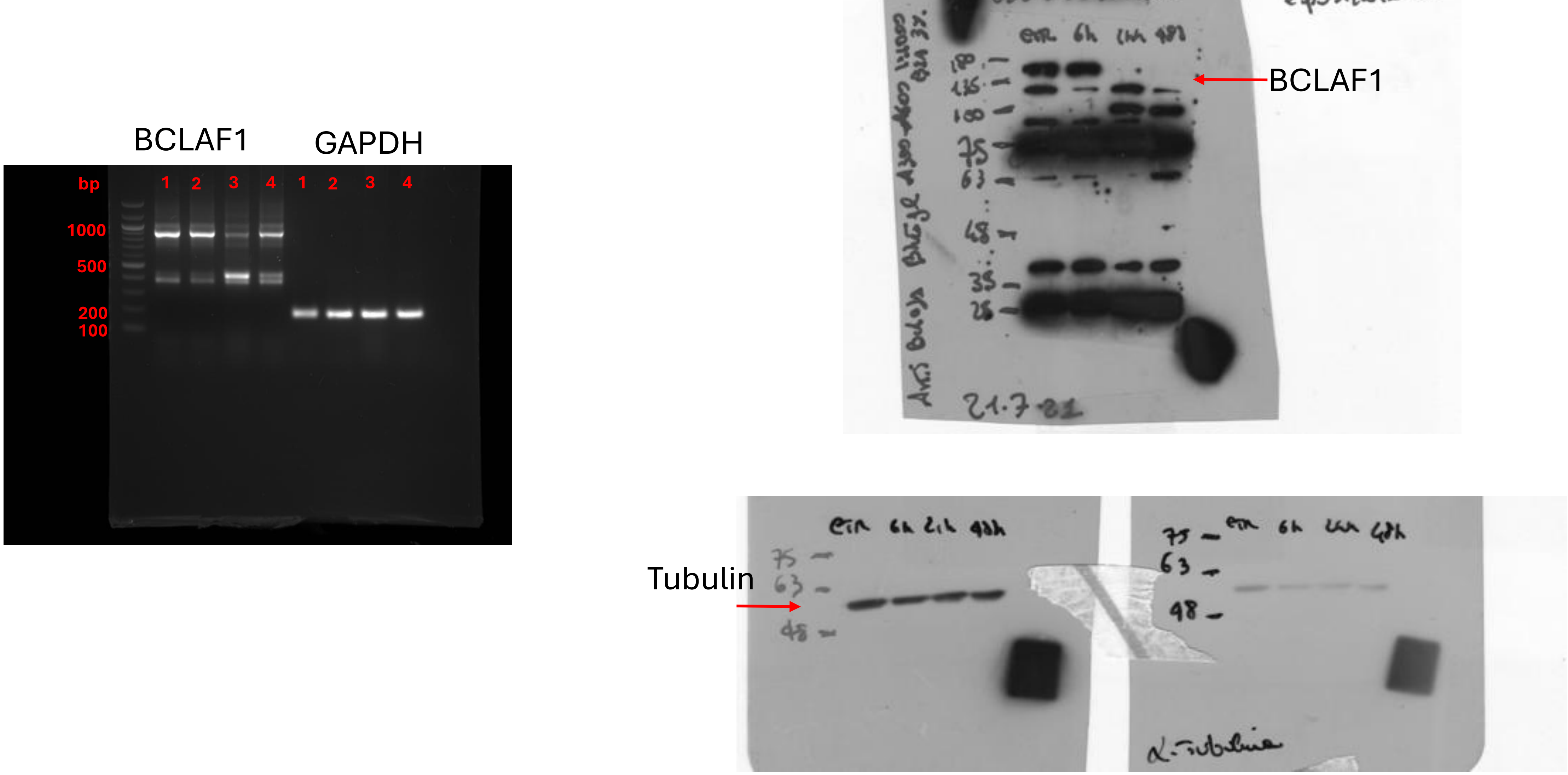

Figure 1D. Left panel: PCR on U937 treated with DMSO (1) 6h SAHA 5μM (2) 24h SAHA 5μM 5μM (3) 48h SAHA (4) for BCLAF1 and GAPDH (same order). Right panel: WB on U937 treated with DMSO (CTR) 6h SAHA 5μM (6h) 24h SAHA 5μM (24h) 48h SAHA (48h) 5μM

FIGURE 1E

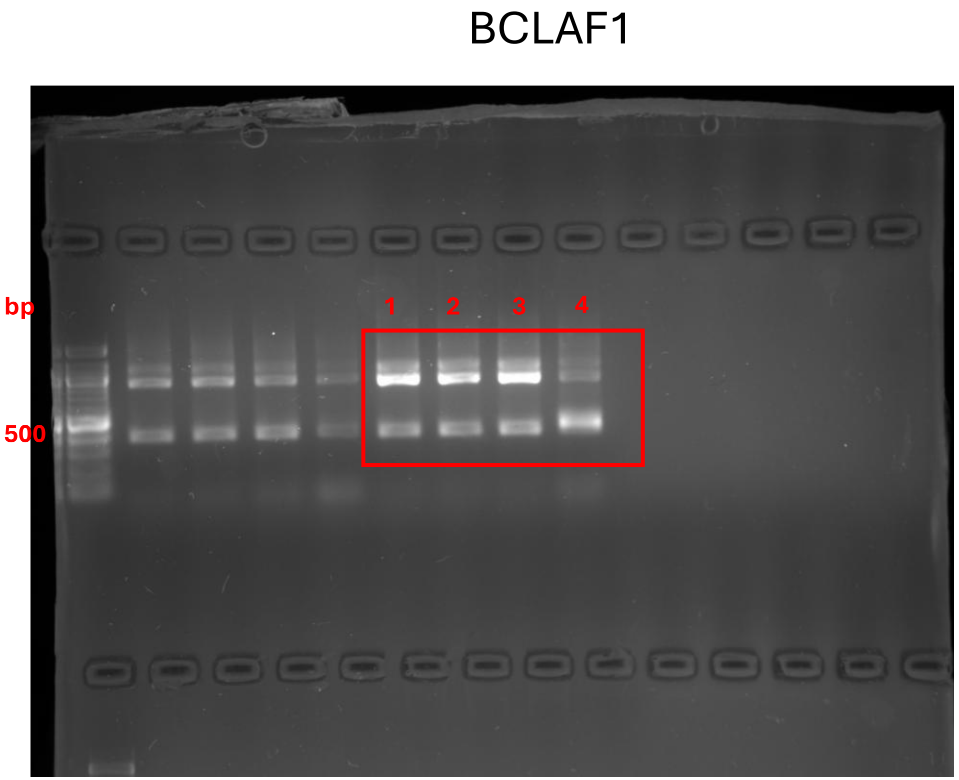

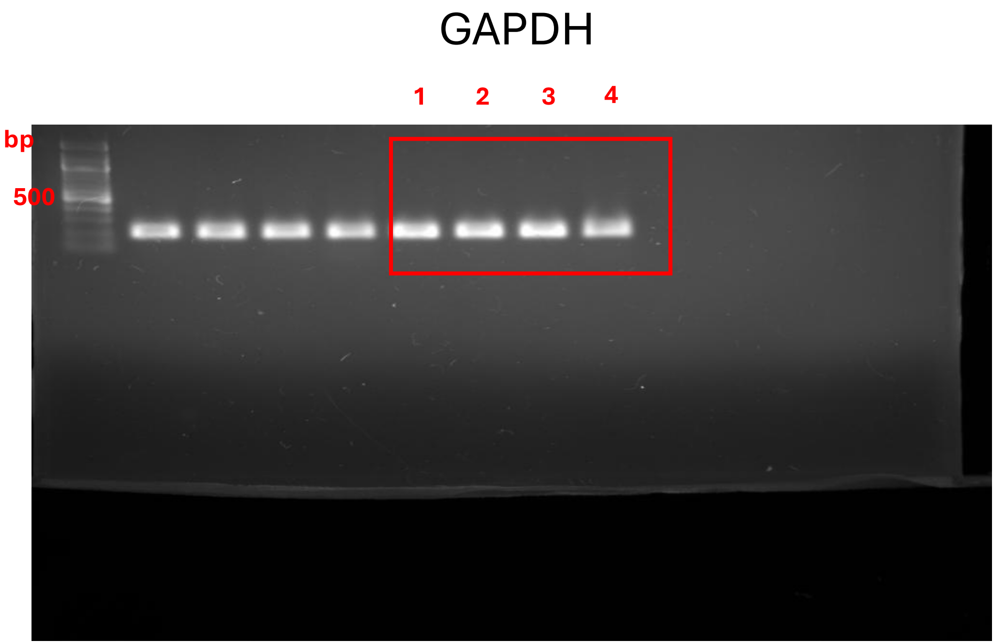

Figure 1E. Left panel: RNA-nascent PCR on U937 treated with DMSO 6h (1) 6h SAHA 5μM (2) 24h DMSO (3) 24h SAHA 5μM (4) for BCLAF1 and GAPDH (same order).

FIGURE 1F

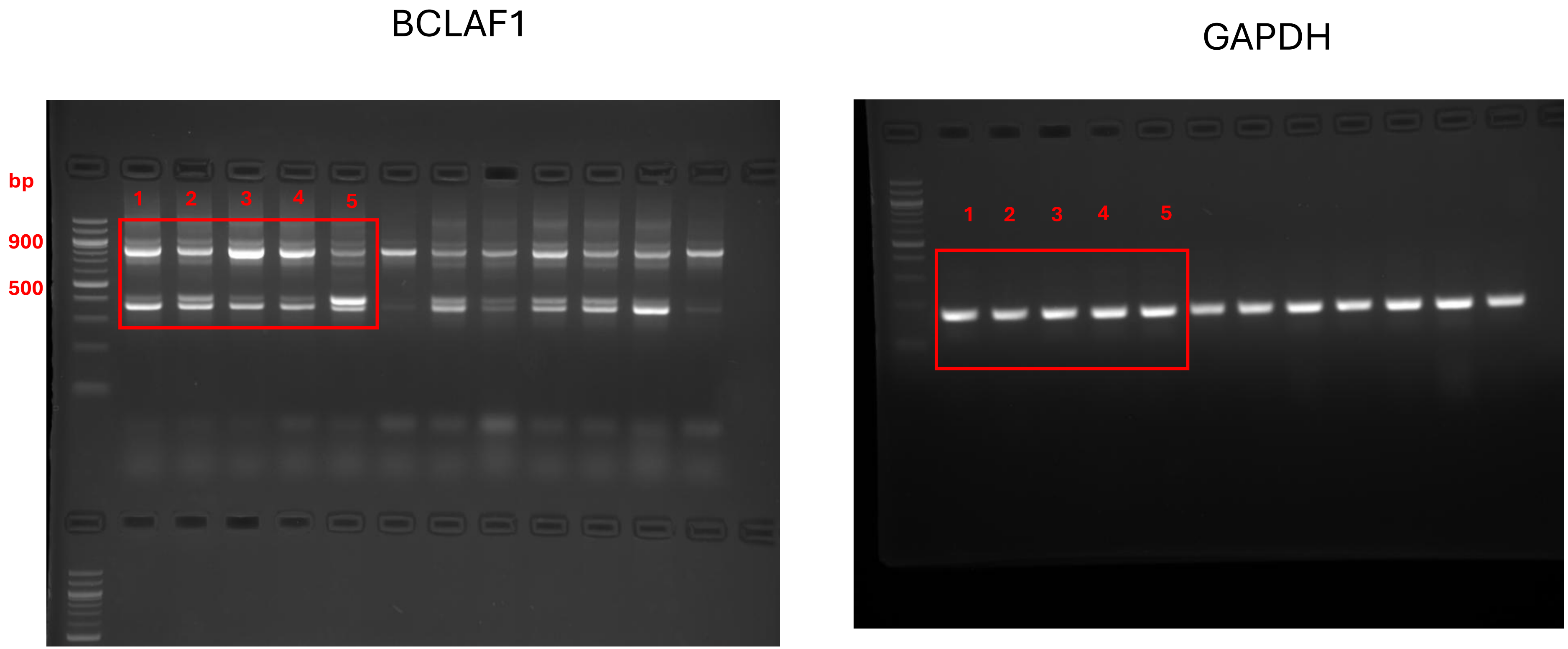

Figure 1F. PCR analysis of BCLAF1 and GAPDH expression in U937 cells treated 24h with DMSO (1) SGI-1027 10 μM (2), EML951 5 μM (3), GSK3685032 10 μM (4), and SAHA 5 μM (5).

FIGURE 2

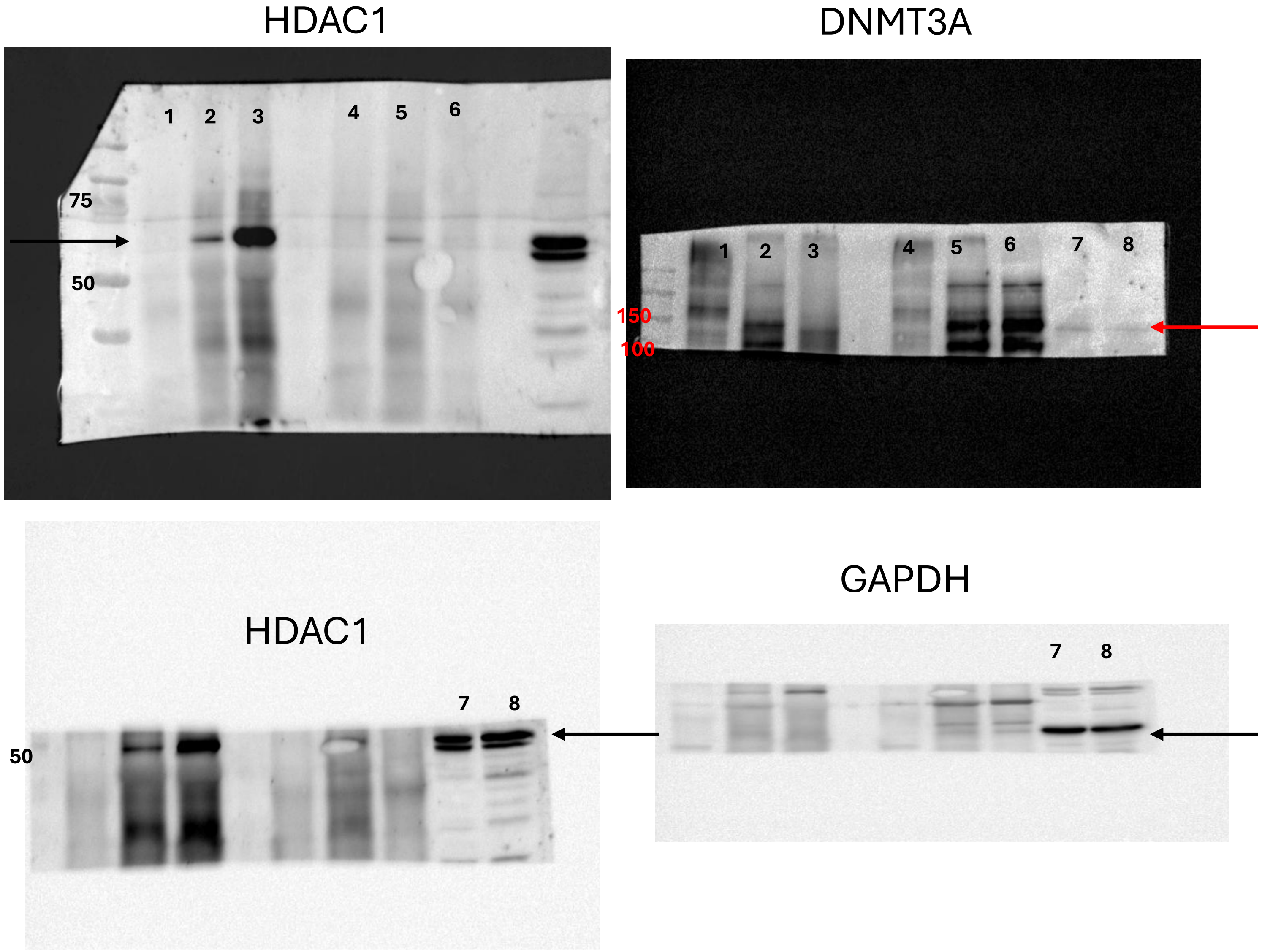

Figure 2C. WB for HDAC1 (left panel) and DNMT3A (right panel) in U937 coimmunoprecipitation: IGG (1) IP HDAC1 CTR (2) IP HDAC1 SAHA 24h 5 μM (3) IGG (4) IP DNMT3A CTR (5) IP DNMT3A SAHA 24h 5 μM (6) INPUT (7) INPUT + SAHA 24h 5 μM (8)

FIGURE 3

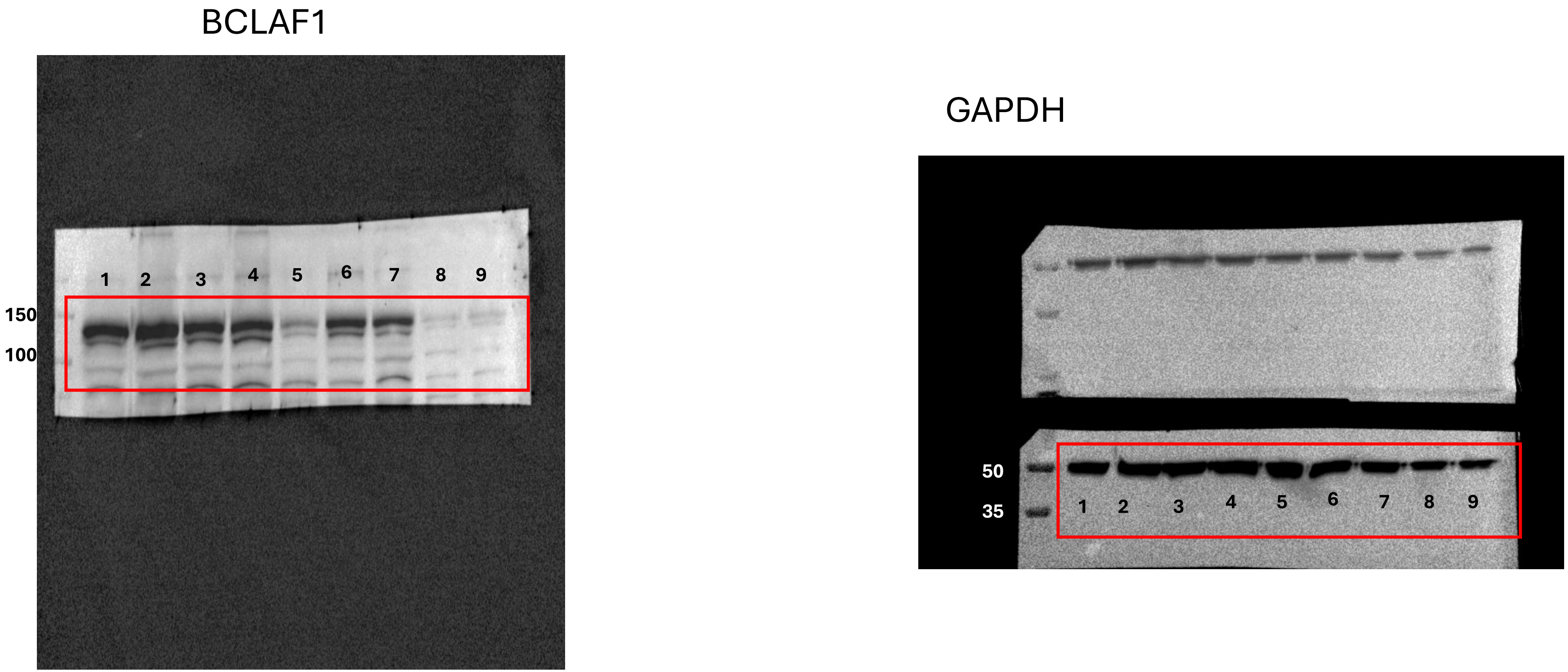

Figure 3B. WB for DNMT3A (left panel) and Tubulin (right panel) in U937 shCTR (1), U937 sh-DNMT3A clones 1C and 2C treated respectively with DMSO (2 and 6), doxycycline 2 µg/mL at 6h (3 and 7), 24h doxycycline 2 µg/mL (4 and 8), and doxycycline 2 µg/mL 48 h (5 and 9)

FIGURE 3

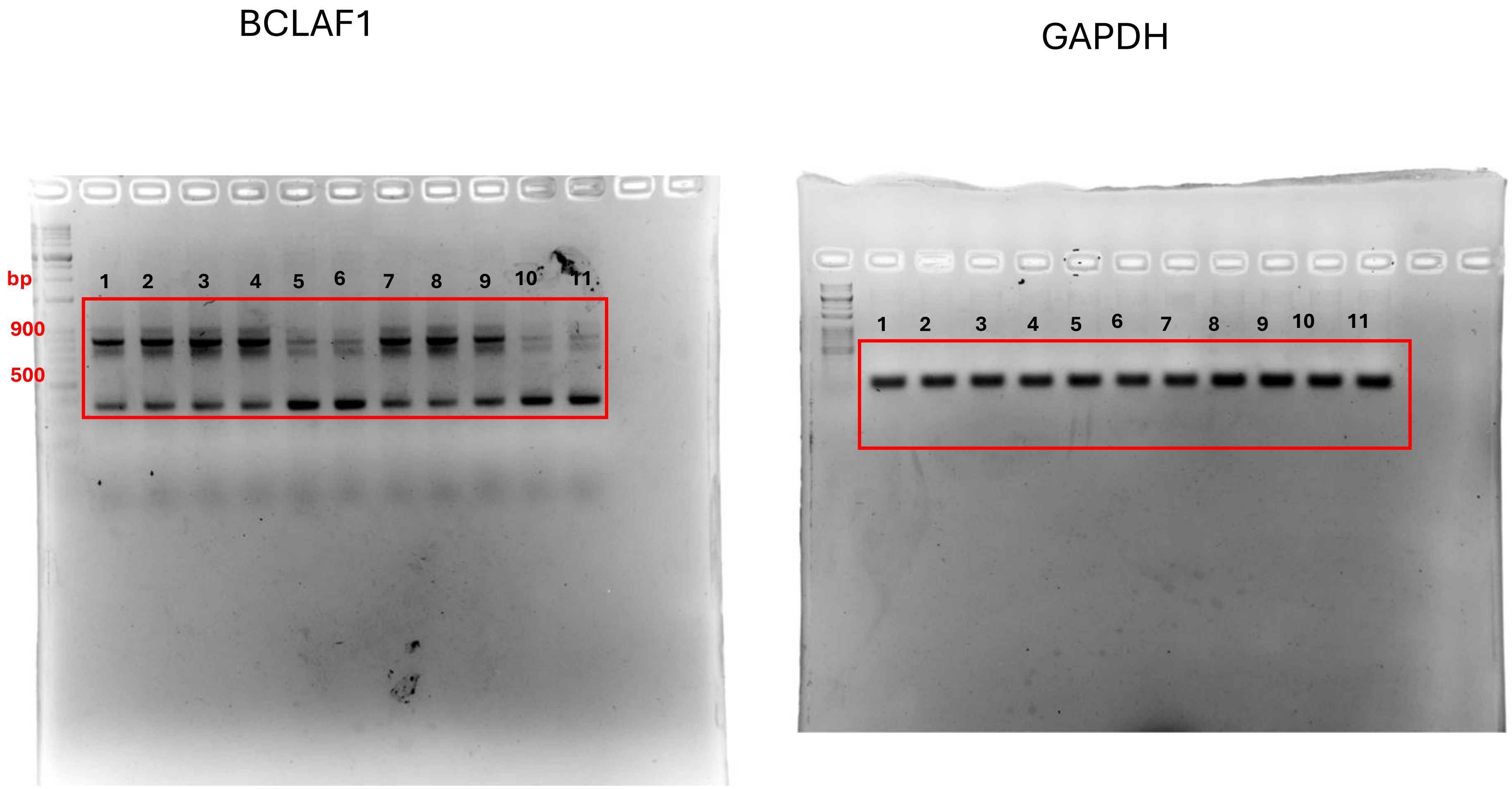

Figure 3D. PCR for BCLAF1 (left panel) and GAPDH (right panel) in U937 wt (1), U937 shCTR (2 and 7), U937 sh-DNMT3A clones 1C and 2C treated respectively with DMSO (3 and 8), doxycycline 2 µg/mL at 6h (4 and 9), 24h doxycycline 2 µg/mL (5 and 10), and doxycycline 2 µg/mL 48 h (6 and 10)

FIGURE 4

DNMT3B

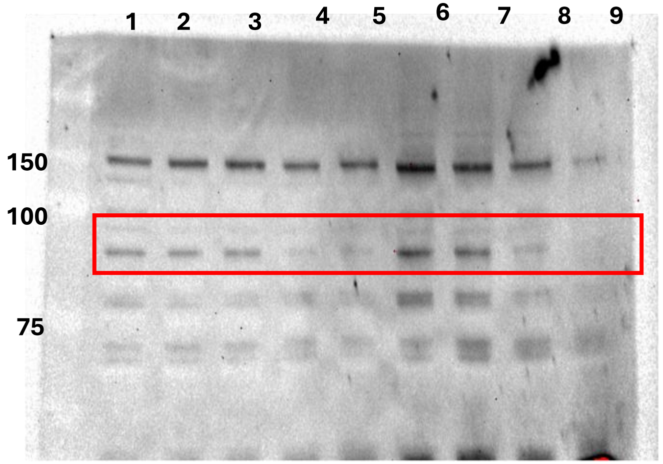

GAPDH

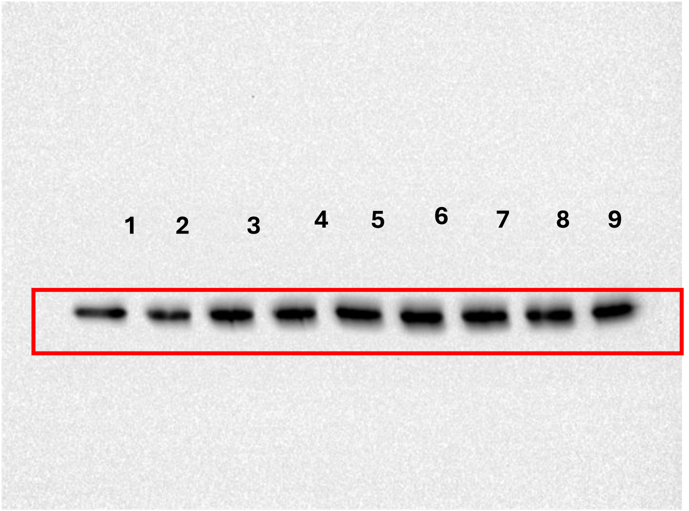

Figure 4B. WB for DNMT3B (left panel) and GAPDH (right panel) in U937 shCTR (1), U937 sh-DNMT3B clones 1D and 2D treated respectively with DMSO (2 and 6), doxycycline 2 µg/mL at 6h (3 and 7), 24h doxycycline 2 µg/mL (4 and 8), and doxycycline 2 µg/mL 48 h (5 and 9)

FIGURE 4

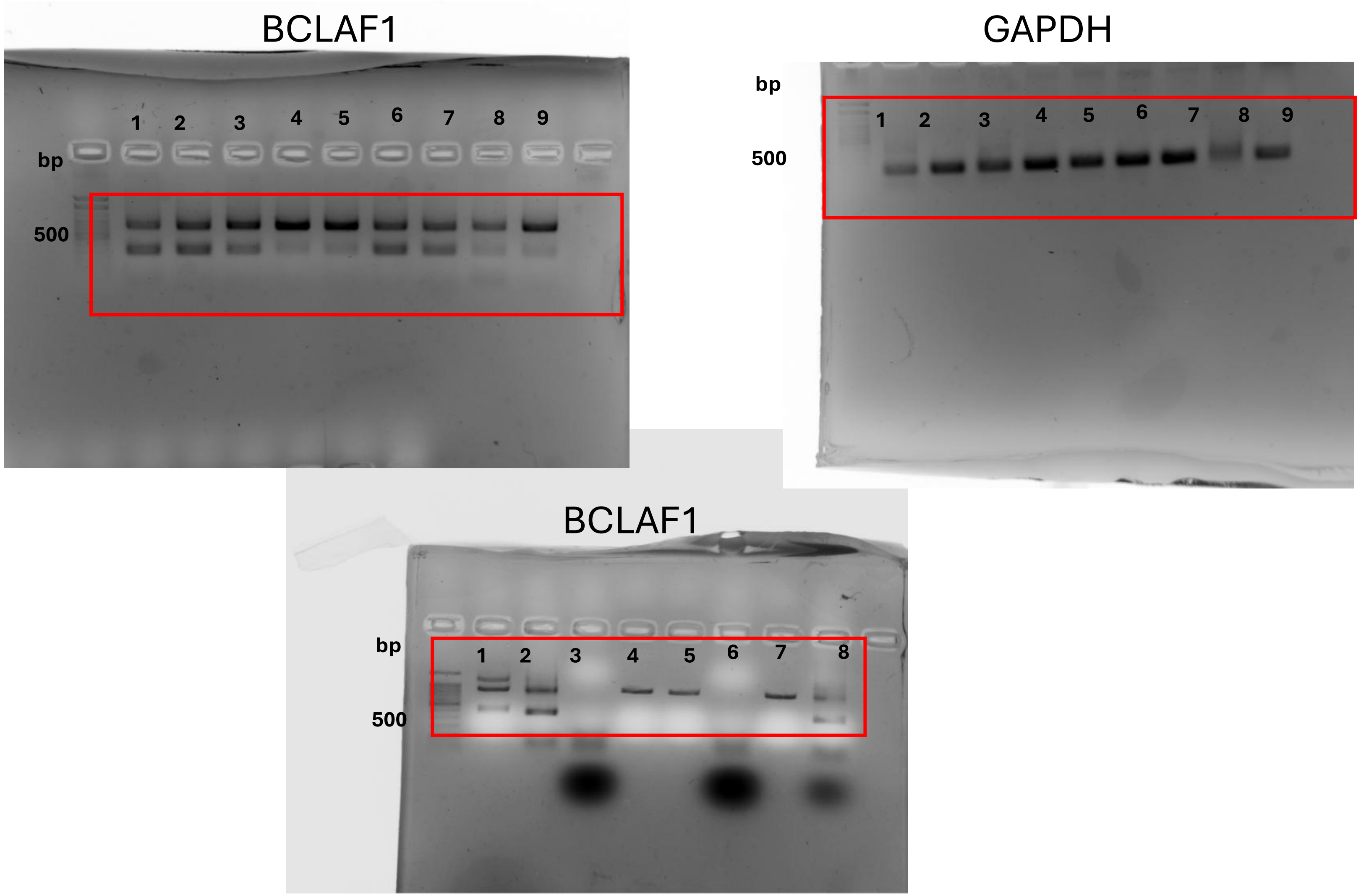

Figure 4D. Upper part: PCR for BCLAF1 (left panel) and GAPDH (right panel) in U937 shCTR (1), U937 sh-DNMT3B clones 1D and 2D treated respectively with DMSO (2 and 6), doxycycline 2 μg/mL at 6h (3 and 7), 24h doxycycline 2 μg/mL (4 and 8), and doxycycline 2 μg/mL 48 h (5 and 9). Lower part: PCR for BCLAF1 in U937 wt cells treated with DMSO (1) and SAHA 5 μM for 24 h (2) and for RIP immunoprecipitating in U937 sh-DNMT3A knockout clones, IGG (3) SRSF10 + DMSO (4), DNMT3B + DMSO (5) and after 48 h of doxycycline (2 μg/mL) for IGG (6), SRSF10 (7) and DNMT3B (8)

FIGURE 5

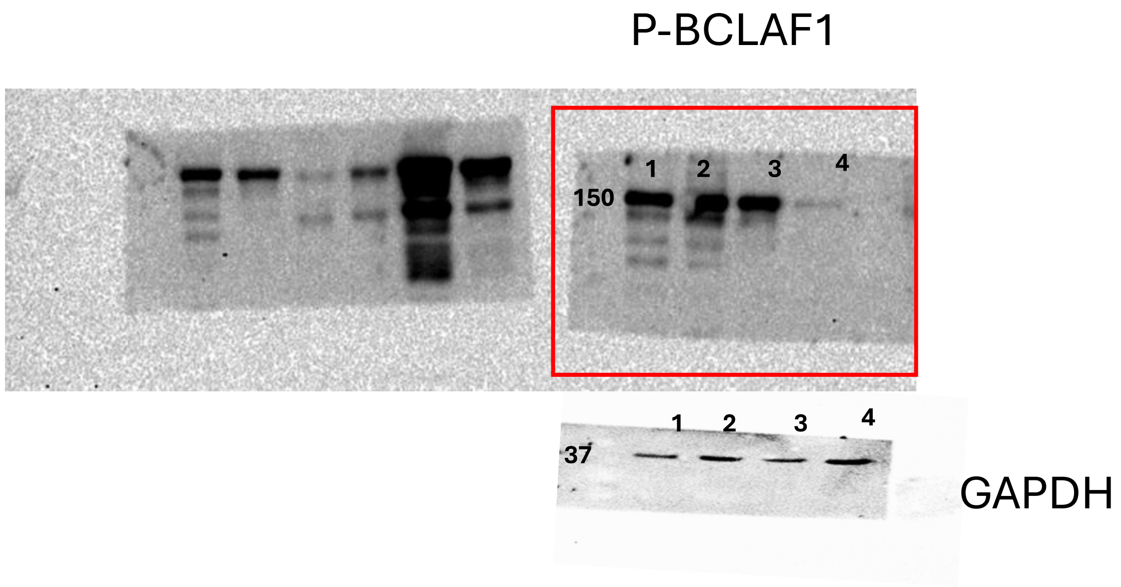

Figure 5A. Upper part: WB for pBCLAF1 (upper panel), GAPDH (lower panel) and H3 (lower panel) in U937 treated with DMSO for 24h (1), SAHA 5 μM 6h (2), SAHA 5 μM 24h (3), SAHA 5 μM 48h (4).

FIGURE 5

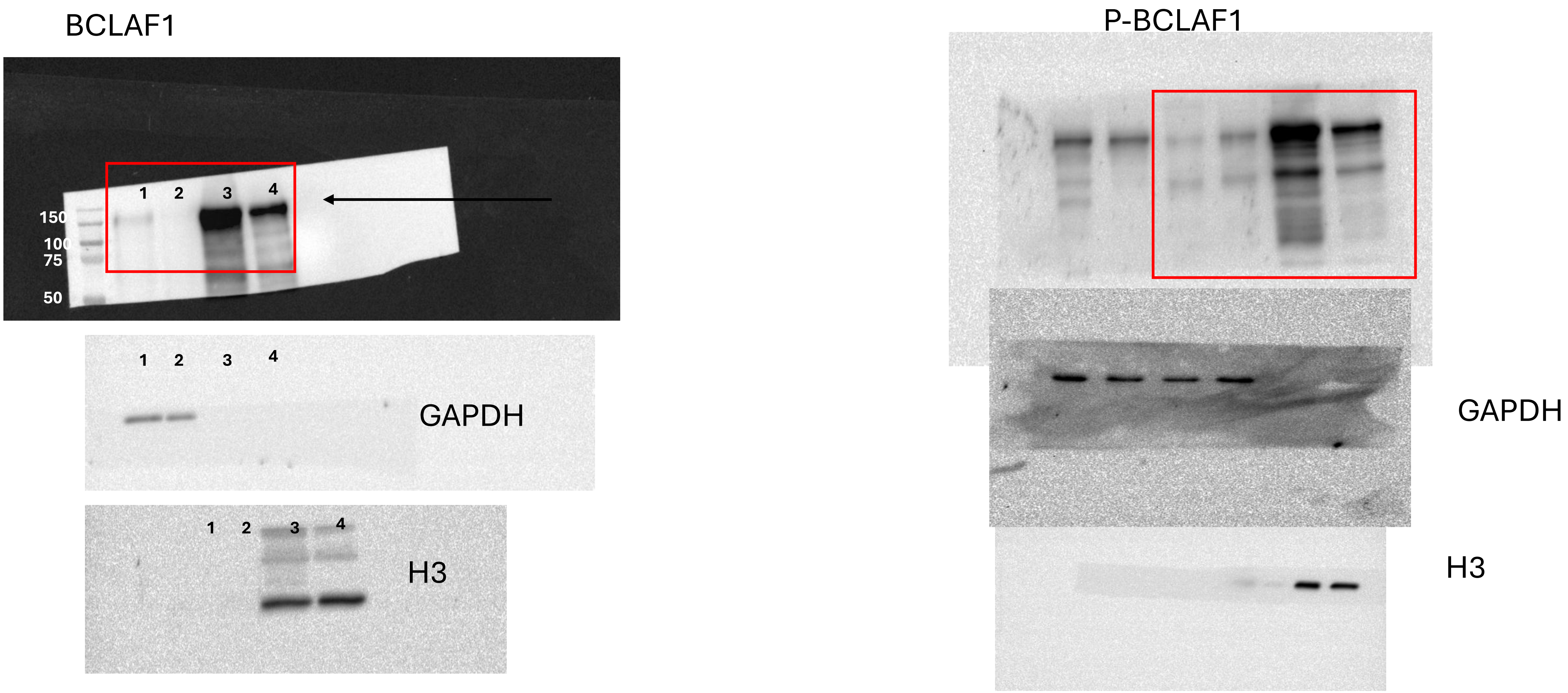

Figure 5B. Upper part: WB for BCLAF1 (upper panel), GAPDH (middle panel) and H3 (lower panel) in U937 nucleus/cytosol extraction treated with DMSO for 24h (1 cit 3 nucl) and SAHA 5 μM 24h (2 cit 4 nucl). Lower part: WB for p-BCLAF1 (upper panel), GAPDH (middle panel) and H3 (lower panel) in U937 nucleus/cytosol extraction treated with DMSO for 24h (1 cit 3 nucl) and SAHA 5 uM 24h (2 cit 4 nucl)

SUPPLEMENTARY FIGURE 1

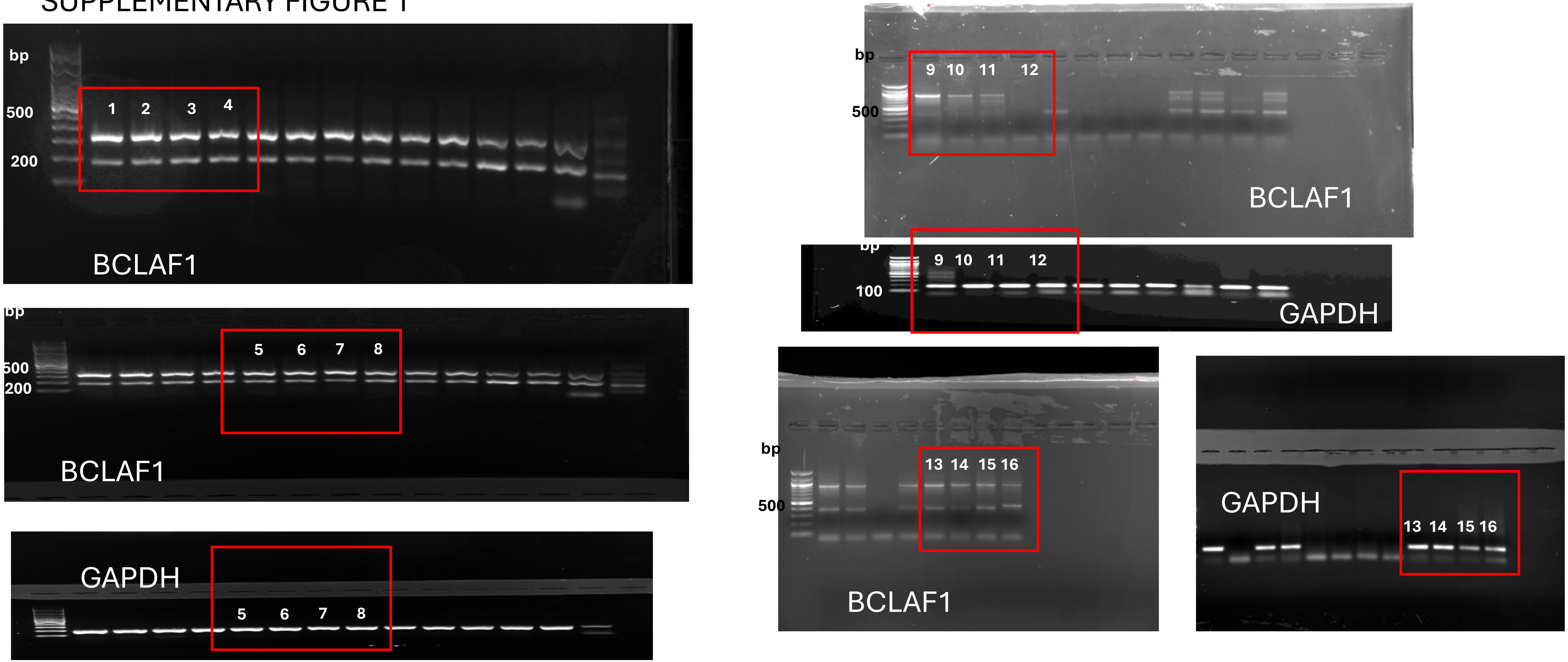

Figure S1B. Left panel upper: PCR for BCLAF1 exon 11 in U937 (1), NB4(2), K562 (3) and CD34+ (4). S1E left panel lower PCR on BCLAF1 exon 11 and GAPDH in U937 treated with DMSO (5), with SAHA 5 μM 6h (6), SAHA 5 μM 24h (7) and SAHA 5 μM 48h (8). S1C Right panel upper: PCR on K562 for BCLAF1 exon5 and GAPDH with with DMSO (9), with SAHA 5 μM 6h (10), SAHA 5 μM 24h (11) and SAHA 5 μM 48h (12). Right panel lower: PCR on NB4 for BCLAF1 exon5 and GAPDH with with DMSO (13), with SAHA 5 μM 6h (14), SAHA 5 μM 24h (15) and SAHA 5 μM 48h (16).

SUPPLEMENTARY FIGURE 1

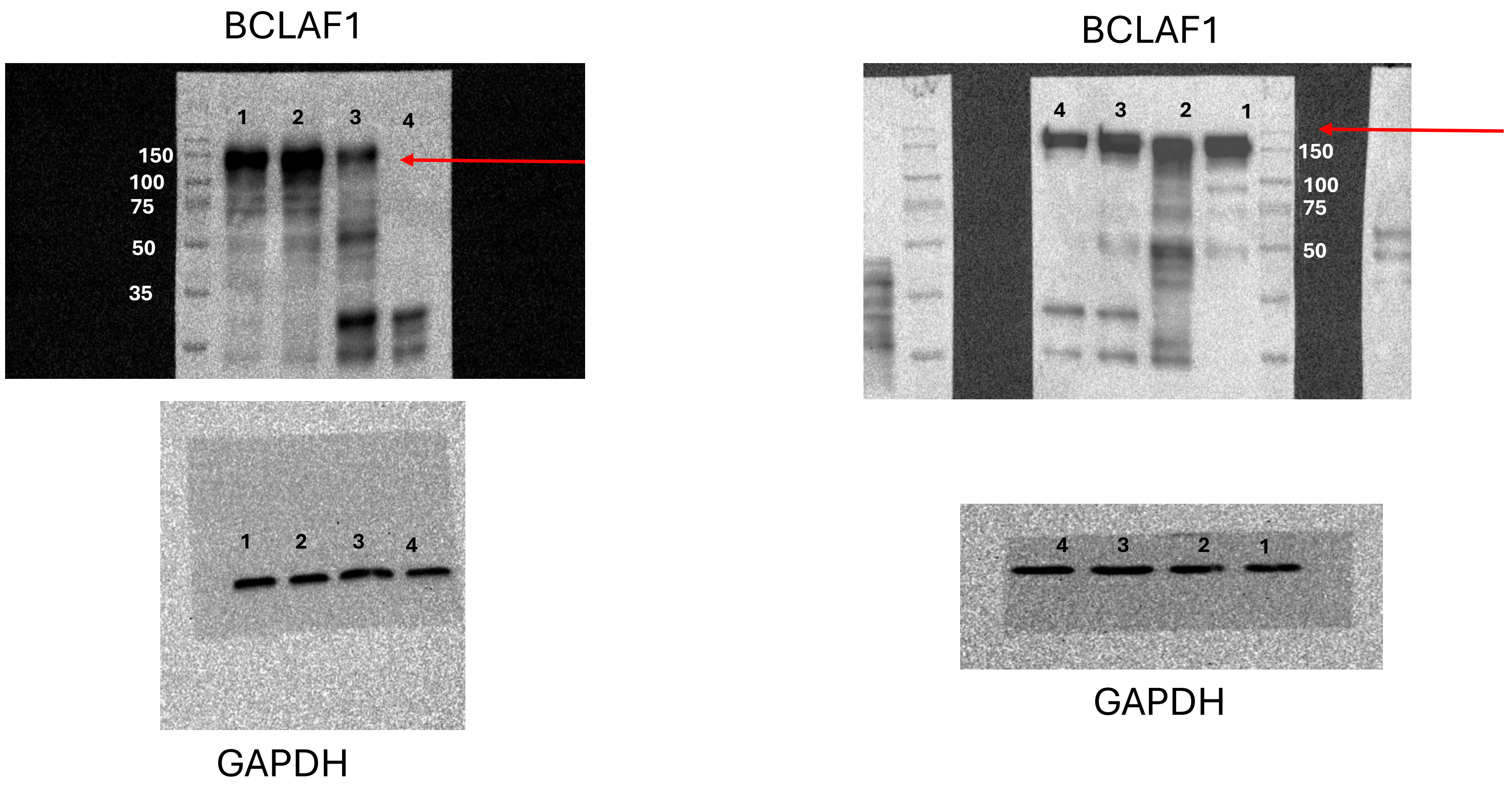

Figure S1C Left Panel: WB on K562 for BCLAF1 exon5 and GAPDH with with DMSO (1), with SAHA 5 μM 6h (2), SAHA 5 μM 24h (3) and SAHA 5 μM 48h (5). Right panel lower: WB on NB4 for BCLAF1 exon5 and GAPDH with with DMSO (6), with SAHA 5 μM 6h (7), SAHA 5 μM 24h (8) and SAHA 5 μM 48h (9).

SUPPLEMENTARY FIGURE 1

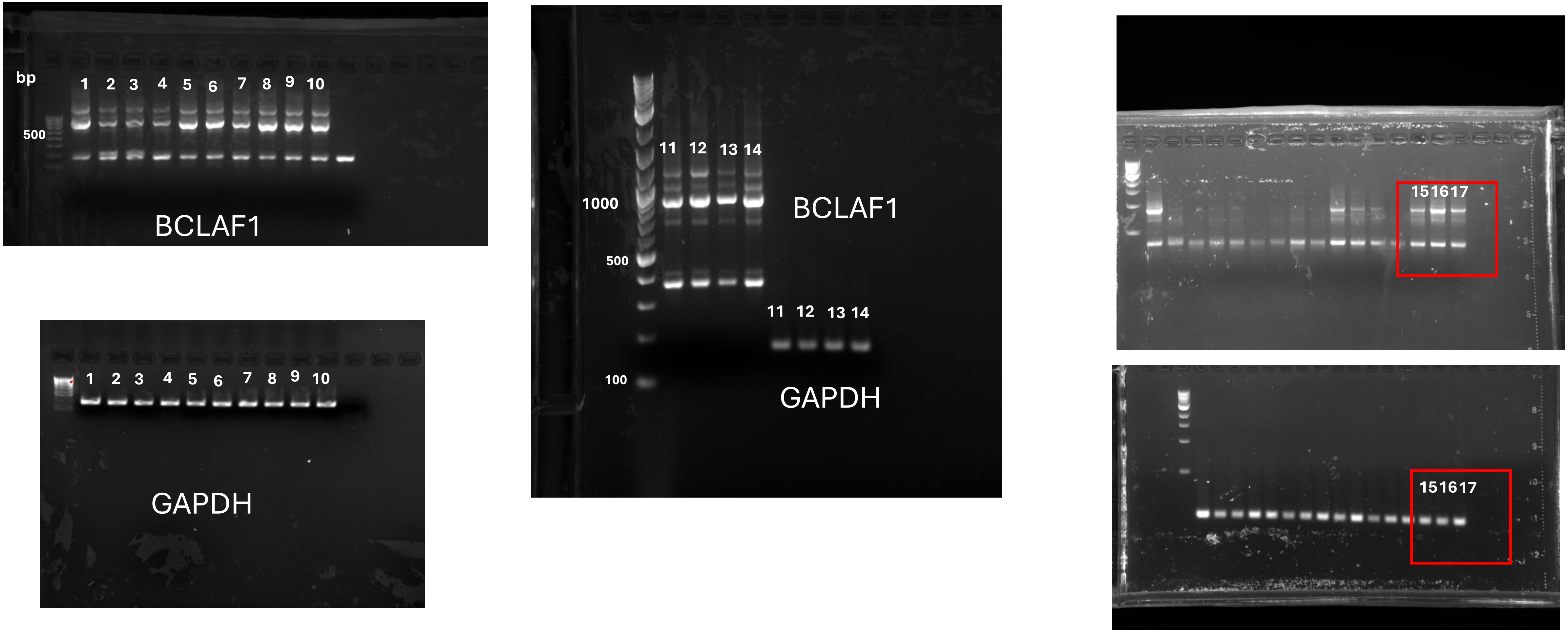

Figure S1F and S1G Left panel : PCR on BCLAF1(upper) and GAPDH (lower) in U937 cells treated for 24 h with DMSO (1), SAHA 5 µM (2), MS-275 5 µM (3), 5-azacytidine 5 µM (4), tasquiminod 5 µM (5), tricostatin A 150 nM (6), EX275 5 µM (7), cisplatin 5 µM (8), and meclofenamic acid 25 and 50 µM (9-10). S1 H Middle panel: PCR on BCLAF1 exon 5 and GAPDH in U937 cells treated for 24 h with DMSO (11) tasquiminod 5 (12) and 50 µM (13) and HDAC6 inhibitor (14). Right panel: PCR for BCLAF1 exon 5 and GAPDH in U937 cells treated for 24 h with santacruzamate 5 µM (15), 10 µM (16), and 50 µM (17)

SUPPLEMENTARY FIGURE 1

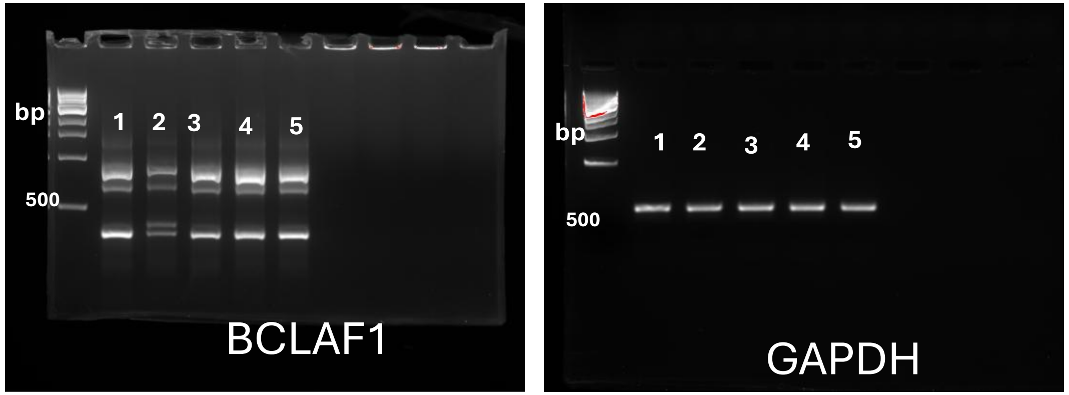

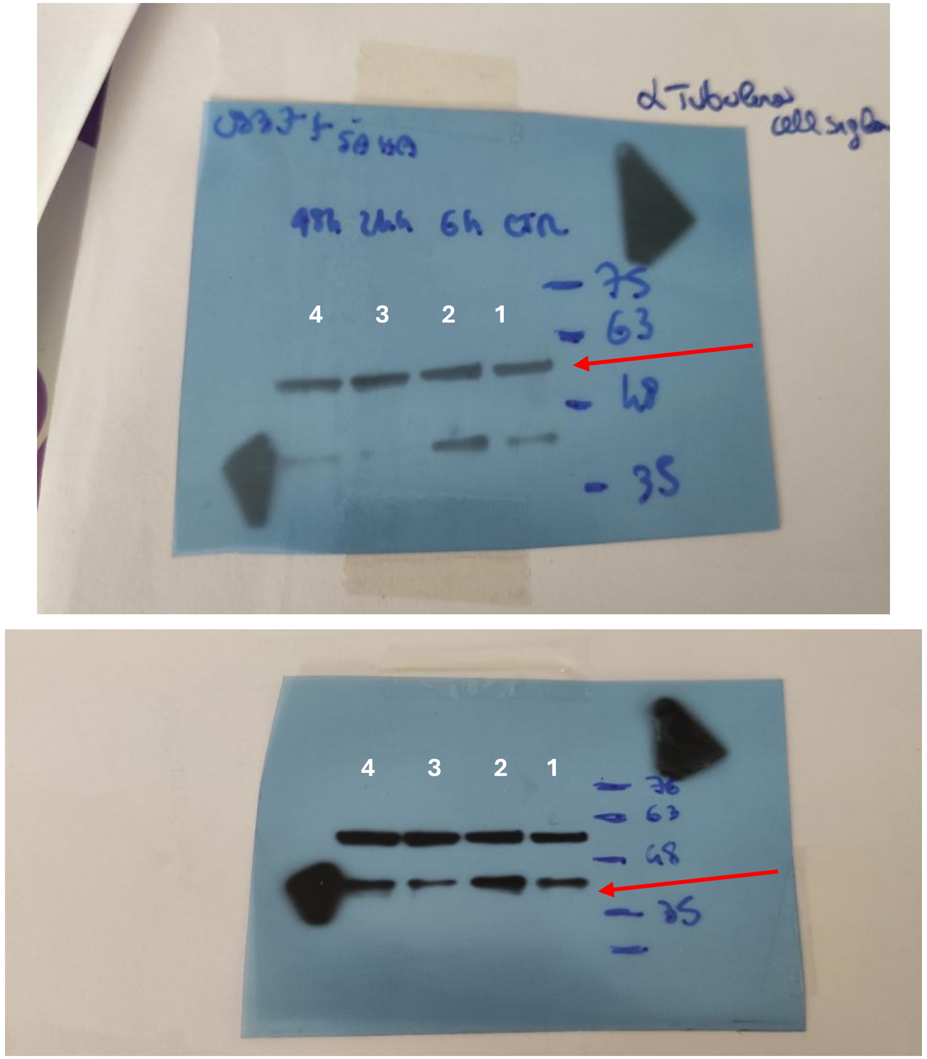

Figure S1I. Left panel upper: PCR for BCLAF1 exon 5 and GAPDH in U937 cells treated for 24 h with DMSO (1) SAHA 5 μM (2) and EML951 5 μM (3), 10 μM (4), and 50 μM (5). Right panel: WB for SRSF10 and Tubulin in U937 with DMSO (1) 6h SAHA 5μM (2) 24h SAHA 5μM 5μM (3) 48h SAHA (4)

SUPPLEMENTARY FIGURE 1

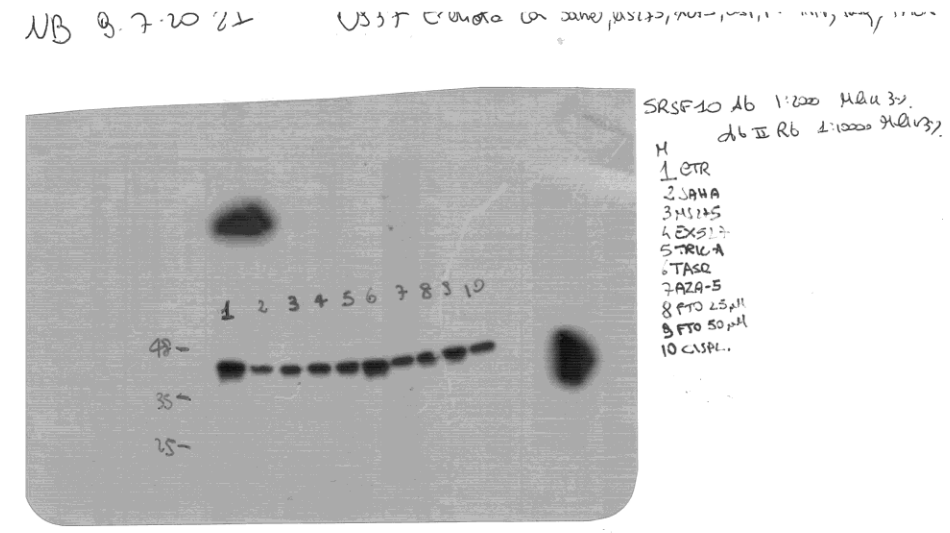

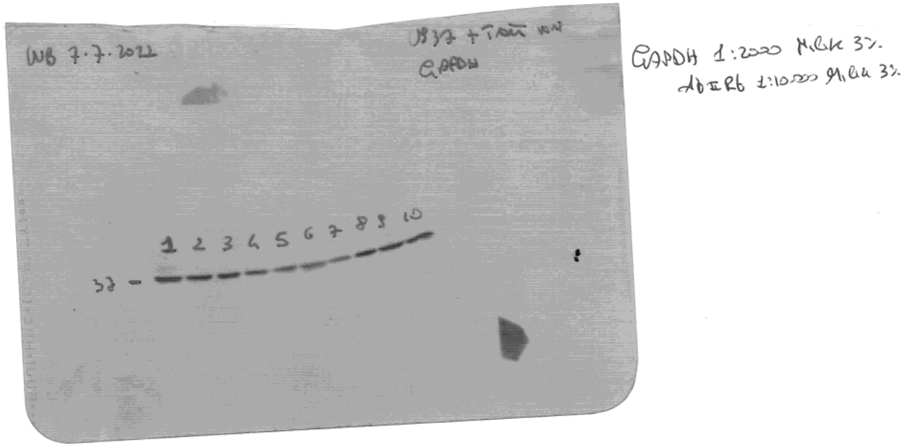

WB on SRSF10 (upper) and GAPDH (lower) in U937 cells treated for 24 h with DMSO (1), SAHA 5 µM (2), MS-275 5 µM (3), 5-azacytidine 5 µM (4), tasquiminod 5 µM (5), tricostatin A 150 nM (6), EX275 5 µM (7), cisplatin 5 µM (8), and meclofenamic acid 25 and 50 µM (9-10)

SUPPLEMENTARY FIGURE 3

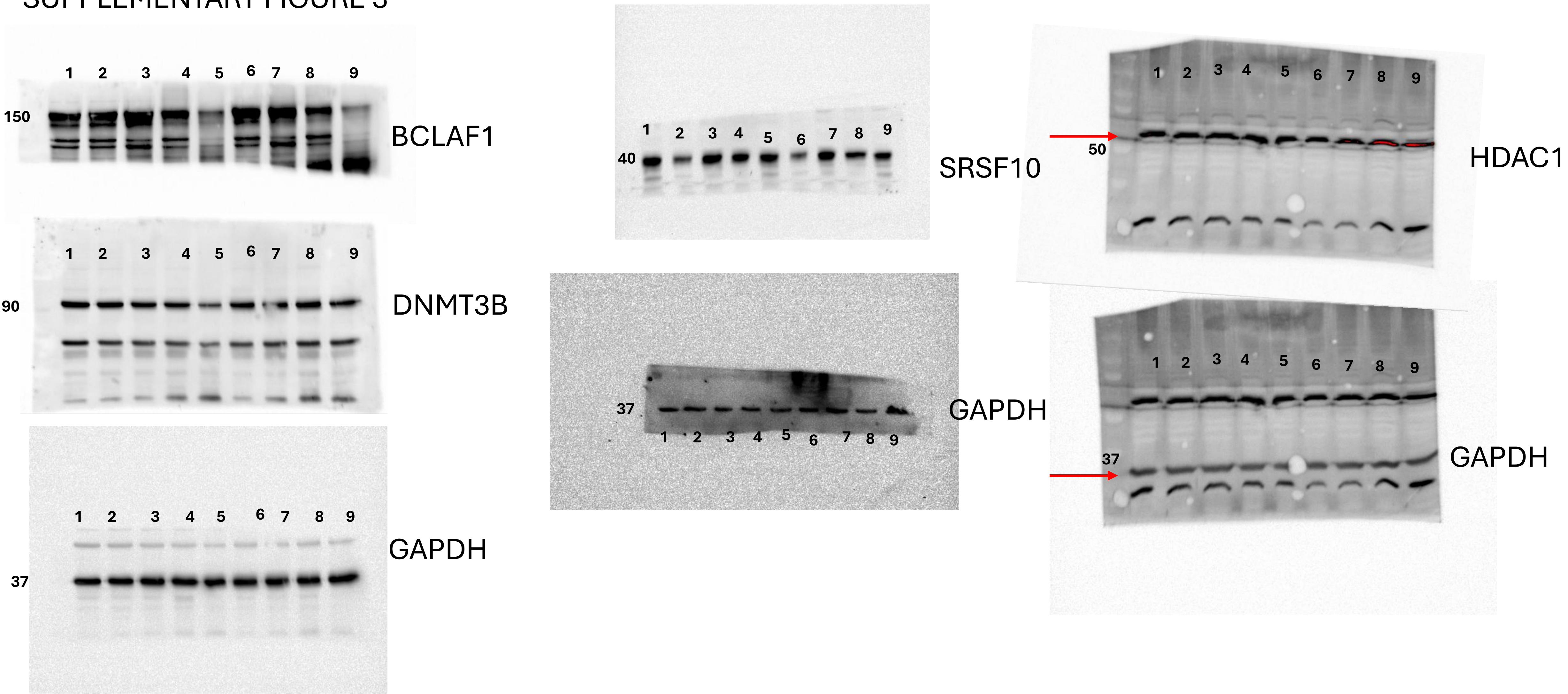

Figure S3B. WB for BCLAF1 (left panel upper), DNMT3B (left panel middle), GAPDH (left panel lower) in U937 shCTR (1), U937 sh-DNMT3A clones 1C and 2C treated respectively with DMSO (2 and 6), doxycycline 2 µg/mL at 6h (3 and 7), 24h doxycycline 2 µg/mL (4 and 8), and doxycycline 2 µg/mL 48 h (5 and 9). WB for SRSF10 (middle panel upper) and GAPDH (middle panel lower) in U937 shCTR (1), U937 sh-DNMT3A clones 1C and 2C treated respectively with DMSO (2 and 6), doxycycline 2 µg/mL at 6h (3 and 7), 24h doxycycline 2 µg/mL (4 and 8), and doxycycline 2 µg/mL 48 h (5 and 9).). WB for HDAC1 (right panel upper) and GAPDH (right panel lower) in U937 shCTR (1), U937 sh-DNMT3A clones 1C and 2C treated respectively with DMSO (2 and 6), doxycycline 2 µg/mL at 6h (3 and 7), 24h doxycycline 2 µg/mL (4 and 8), and doxycycline 2 µg/mL 48 h (5 and 9).

SUPPLEMENTARY FIGURE 4

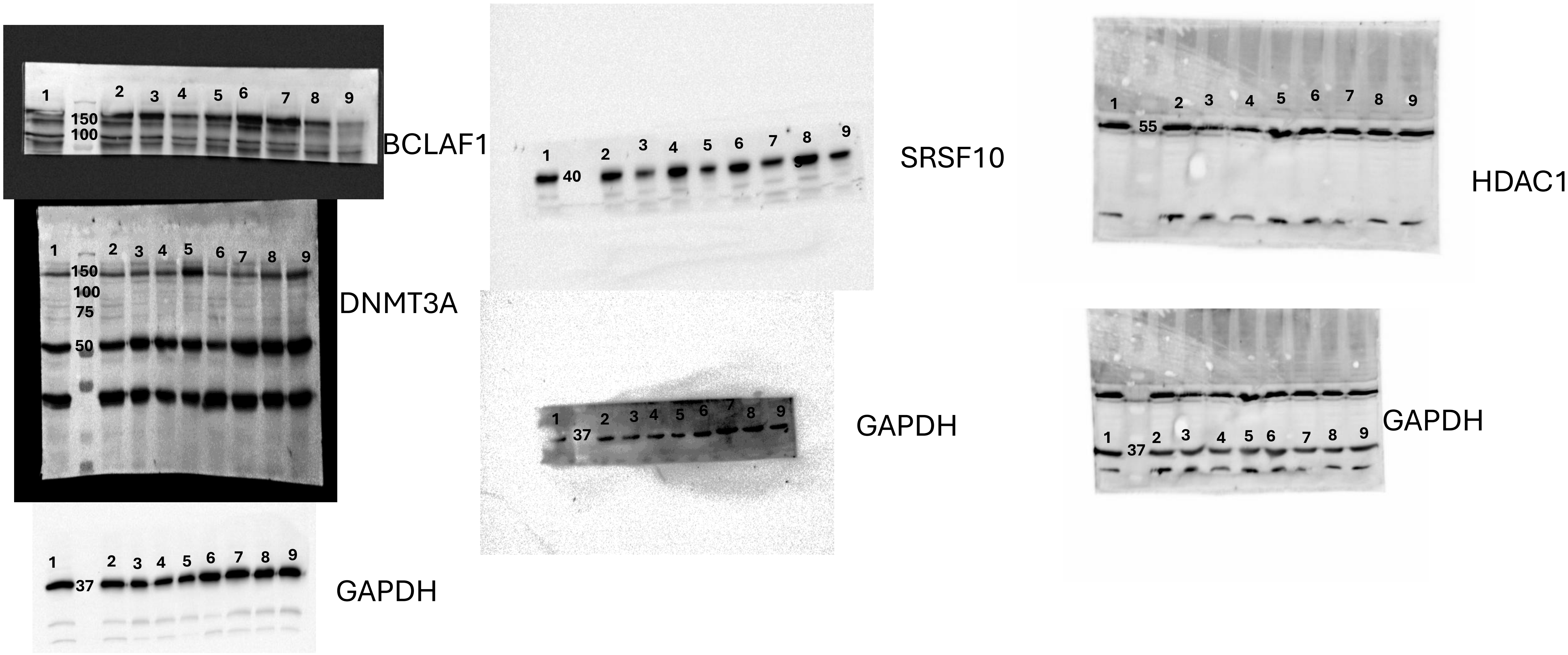

Figure S4B. WB for BCLAF1 (left panel upper), DNMT3A (left panel middle), GAPDH (left panel lower) in U937 shCTR (1), U937 sh-DNMT3B clones 1D and 2D treated respectively with DMSO (2 and 6), doxycycline 2 µg/mL at 6h (3 and 7), 24h doxycycline 2 µg/mL (4 and 8), and doxycycline 2 µg/mL 48 h (5 and 9). WB for SRSF10 (middle panel upper) and GAPDH (middle panel lower) in U937 shCTR (1), U937 sh-DNMT3A clones 1C and 2C treated respectively with DMSO (2 and 6), doxycycline 2 µg/mL at 6h (3 and 7), 24h doxycycline 2 µg/mL (4 and 8), and doxycycline 2 µg/mL 48 h (5 and 9).). WB for HDAC1 (right panel upper) and GAPDH (right panel lower) in U937 shCTR (1), U937 sh-DNMT3A clones 1C and 2C treated respectively with DMSO (2 and 6), doxycycline 2 µg/mL at 6h (3 and 7), 24h doxycycline 2 µg/mL (4 and 8), and doxycycline 2 µg/mL 48 h (5 and 9).

SUPPLEMENTARY FIGURE 4

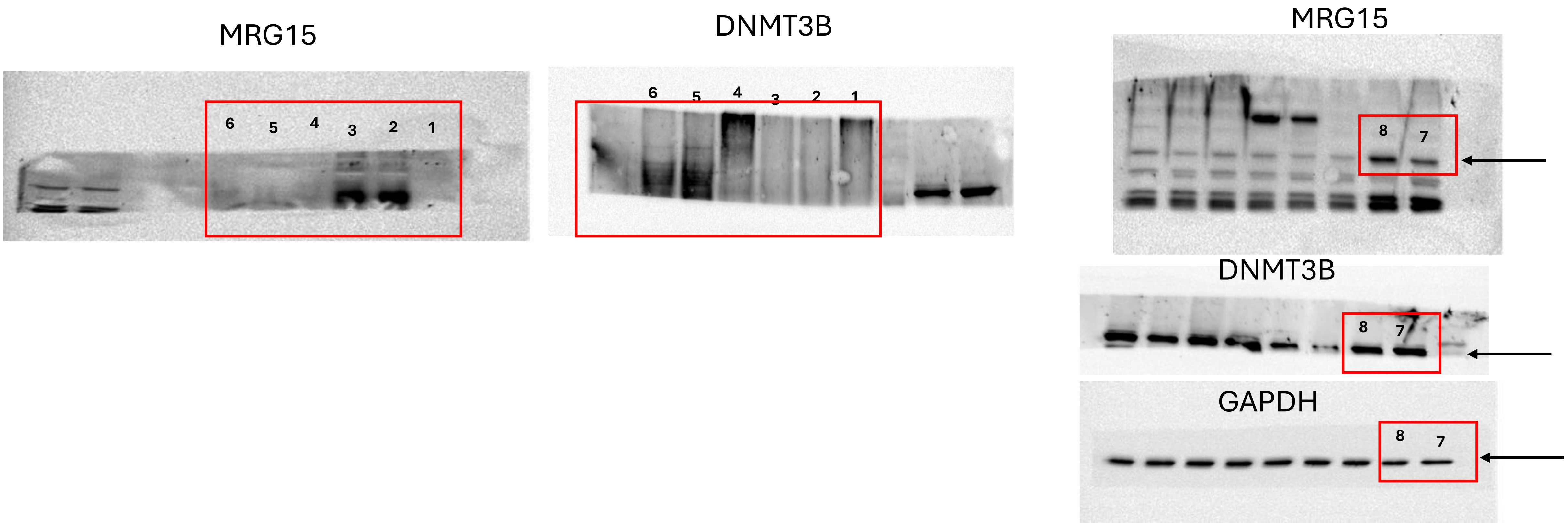

Figure S4C. WB for MRG15 (left panel) and DNMT3B (right panel) in U937 coimmunoprecipitation: IGG (1) IP MRG15 CTR (2) IP MRG15 SAHA 24h 5 µM (3) IGG (4) IP DNMT3B CTR (5) IP DNMT3B SAHA 24h 5 µM (6) INPUT (7) INPUT + SAHA 24h 5 µM (8)

SUPPLEMENTARY FIGURE 5

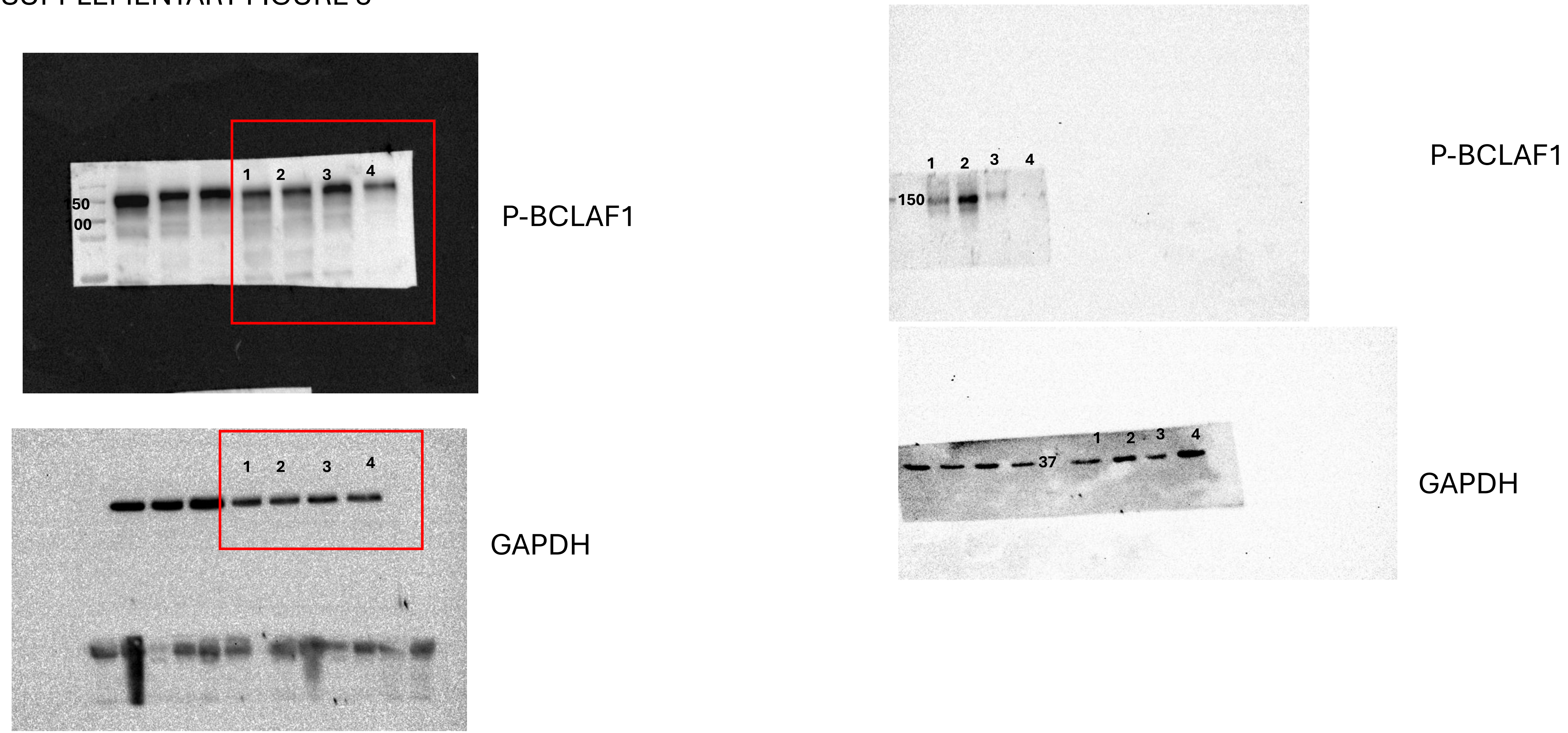

Figure 5B. Left panel: WB on K562 for p-BCLAF1 and GAPDH treated with DMSO (1) 6h SAHA 5μM (2) 24h SAHA 5μM (3) 48h SAHA (4) 5μM. Right panel: WB on NB4 for p-BCLAF1 and GAPDH treated with DMSO (1) 6h SAHA 5μM (2) 24h SAHA 5μM (3) 48h SAHA (4) 5μM

SUPPLEMENTARY FIGURE 5

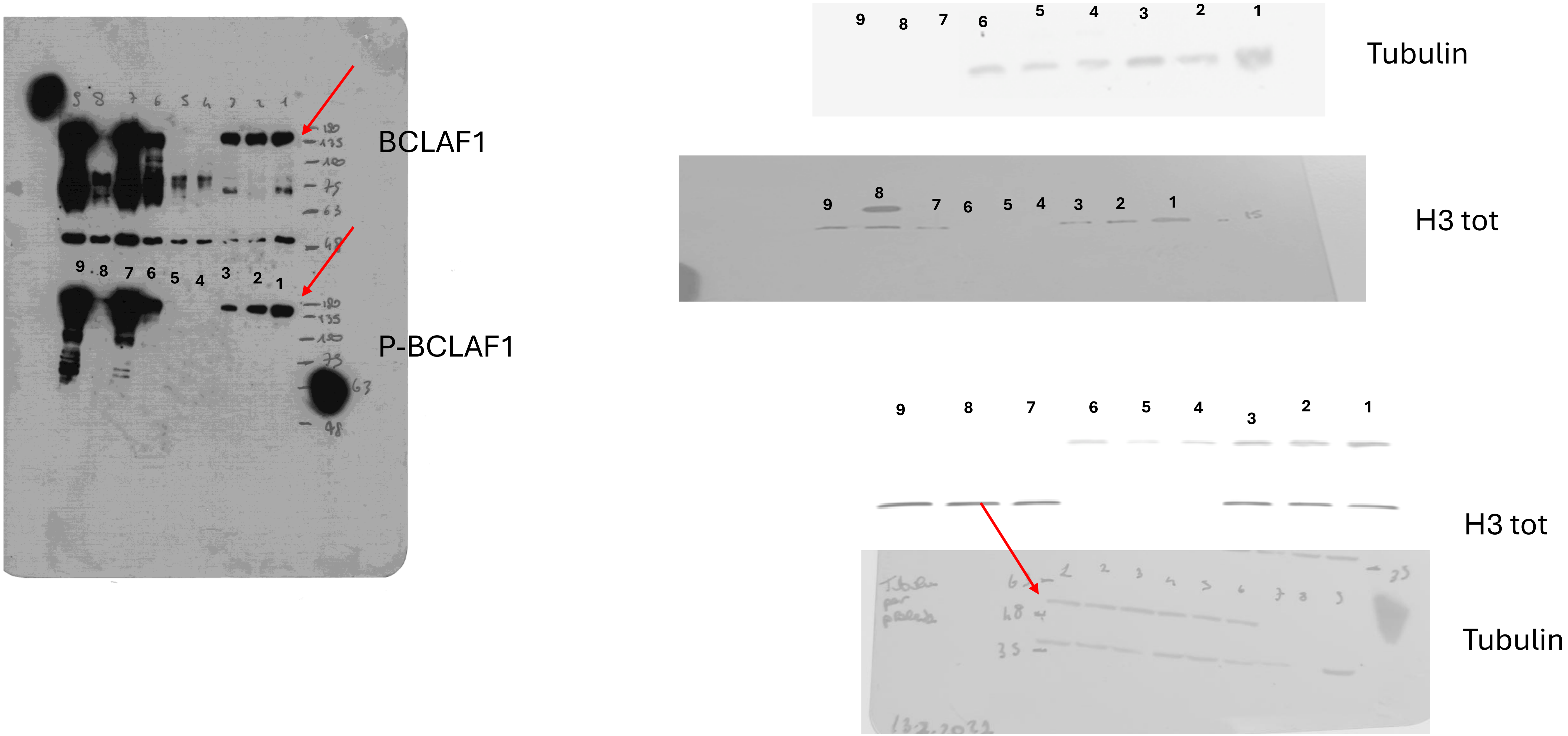

Figure S5C. WB nucleus/citosol for BCLAF1 and in U937 after treatment with SAHA 5 μM and MS275 5 μM at 24h: Upper panel BCLAF1 in U937 DMSO total (1) SAHA total (2) MS275 total (3) DMSO cytosol (4) SAHA cytosol (5) MS275 cytosol (6) DMSO nucleus (7) SAHA nucleus (8) MS275 nucleus (9). Lower panel p-BCLAF1 in U937 DMSO total (1) SAHA total (2) MS275 total (3) DMSO cytosol (4) SAHA cytosol (5) MS275 cytosol (6) DMSO nucleus (7) SAHA nucleus (8) MS275 nucleus (9). Right panel upper H3 and Tubulin for p-BCLAF1; middle panel H3 and tubulinfor BCLAF1
