# Supplementary material for: Dynamic epigenetic regulation of BCLAF1 splicing in acute myeloid leukemia
Source: Cell Death Dis. 2026 Mar 24;17(1):344. doi: 10.1038/s41419-026-08594-4 (PMC13039510; doi:10.1038/s41419-026-08594-4)
Supplement: Supplementary file 4 — Data Set 2 [file 41419_2026_8594_MOESM4_ESM.pdf]

Data repository details:
Project Name: Characterization of U937 cells after SAHA treatment
Project accession: PXD063064
Project DOI: Not applicable

Reviewer access details
Log in to the PRIDE website using the following details:
Project accession: PXD063064
Token: T1fycE0Ipq04

Alternatively, reviewer can access the dataset by logging in to the PRIDE website using the following account details:
Username: reviewer_pxd063064@ebi.ac.uk
Password: CEZwnTxy7IdT
